# Geography and language divergence: The case of Andic languages

**DOI:** 10.1371/journal.pone.0265460

**Published:** 2022-05-26

**Authors:** Ezequiel Koile, Ilia Chechuro, George Moroz, Michael Daniel

**Affiliations:** 1 Linguistic Convergence Laboratory, HSE University, Moscow, Russia; 2 Department of Linguistic and Cultural Evolution, Max Planck Institute for Evolutionary Anthropology, Leipzig, Germany; Leiden University, GERMANY

## Abstract

We study the correlation between phylogenetic and geographic distances for the languages of the Andic branch of the East Caucasian (Nakh-Daghestanian) language family. For several alternative phylogenies, we find that geographic distances correlate with linguistic divergence. Notably, qualitative classifications show a better fit with geography than cognacy-based phylogenies. We interpret this result as follows: The better fit may be due to implicit geographic bias in qualitative classifications. We conclude that approaches to classification other than those based on cognacy run a risk to implicitly include geography and geography-related factors as one basis of genealogical classifications.

## 1. Introduction

Linguistic divergence usually happens in parallel to population splits. It is logical, then, to propose that geographic distances may correlate with linguistic diversity. One typical scenario is that of a dialect continuum, where geographic distance is closely correlated with linguistic diversification [1]. It is also well known that, in many areas of high language density, linguistic varieties that are more closely related may be separated from each other by more distantly related or even unrelated languages, for a variety of historical reasons, for example, cf. maps 106–114 for Eastern Siberia in [2], or the discussions of the observed language distributions in Amazonia in [3,4]. The situation is further complicated by the phenomenon of linguistic convergence, by which two genealogically distant or unrelated varieties that are in contact may show more similarities in some respects than their genealogically closer relatives [5:89–90].

In this paper, we test the correlation between geographic distances and different phylogenies proposed for the languages of the Andic branch of East Caucasian (Nakh-Daghestanian). We obtain a positive correlation for all phylogenies in our dataset. Notably, however, quantitative phylogenies based on lexical cognacy of properly curated basic vocabulary (e.g. removing known loanwords), generally believed to be resistant to the effects of language contact, deliver levels of correlation lower than those obtained from classifications based on qualitative similarities and selected isoglosses. We suggest that the better fit between geography and linguistic classifications that are less immune to convergence arises because geographic adjacency is translated into an implicit geographic bias of qualitative classifications. We conclude that the higher correlation is in fact in the eye of the beholder.

The conventional set of language-internal tools used for phylogenetic reconstruction in historical linguistics includes the analysis of regular sound changes in basic lexicon, comparison of cognate retention and innovation, and comparative morphology and phonology. This list is constantly expanding with the most recent studies e.g. incorporating an assessment of probabilities of certain paths of semantic evolution [6] or systematic comparison of syntactic features [7].

This set of linguistic data is often accompanied by extra-linguistic information, the most important coming from archaeology, population genetics, and geography. Extensive amounts of data from these three sources have been thoroughly analyzed for the languages (and population groups) of Europe, and impressive results regarding the co-evolution of genes and languages have been obtained (see [8–10] for genetics, [11] for archaeology). Similar approaches have also been successfully applied in less studied language families and areas ([12–15]). However, the extra-linguistic nature of these data does not allow to directly link them to linguistic evidence (cf. [16: 23–25], [14:39–30]). Provided that a match between non-linguistic evidence on the one side and linguistic data on the other may be confounded by language shifts and contact phenomena, there are potential caveats regarding these methods.

The practice of proposing hypotheses of linguistic reconstruction that are biased towards geography dates back to the early days of the comparative method with, for example, Dutch linguist Hendrik Kern exercising it in his 1889 reconstruction of Austronesian homeland [17]. Although Kern’s reconstruction was criticized for assigning cognate sets based on geographic distribution rather than linguistic subgrouping [18] and eventually abandoned in subsequent research, the geographic basis in linguistic reconstruction persists.

It appears that some later approaches to the classification of Austronesian languages may have had a geographic bias, such as Western Malayo-Polynesian grouping by Blust [19], which was subsequently rejected [20:435]. Wichmann and Rama [21: Sections 4.5.1–4.5.4] discuss more cases where various proposed subgroups in the Pacific are geographically circumscribed but not sufficiently supported by lexical evidence (South Sulawesi, Central Malayo-Polynesian, South Halmahera-West New Guinea).

A paradigmatic case is Malcolm Guthrie’s classification of Bantu languages, one of the very few explicitly based on a geographic partition of the area inhabited by the speakers of these languages. Although the author called this an “essentially tentative” classification [22: 5], it has had a great impact on comparative research into these languages, to the extent that it is used as part of Bantu languages’ naming system to our days, in certain contexts.

In many other cases one can suspect that the main—even if implicit—reason behind conducting a comparison of phyla or languages is geographic adjacency. For Australian languages, there have been two memorable attempts to unite the Pama-Nyungan and the Non-Pama-Nyungan languages of Australia into one single family. Evans [23] proposed a Macro-Pama-Nyungan family tree, although each node was defined by a single shared innovation. An alternative tree proposed by Heath [24] is based predominantly on pronominal data. In a re-examination by Bowern [25], the evidence for both hypotheses was shown to be inconclusive, suggesting that linguistic data are insufficient. One is justified to suspect that the putative macro-family is implicitly based on extralinguistic considerations, such as merely belonging to the same continent.

Similarly, the "Sepik-Ramu phylum" has been proposed for the languages in the Sepik Ramu basin in Papua-New Guinea [26]. A more recent analysis [27] further divided these languages into two families: Lower Sepik-Ramu, and Sepik. Further attempts to build a coherent classification based on basic lexicon have led to negative results in Wichmann [28: 318], and the original author of this classification, while citing some scattered morphological evidence in support of this claim, admitted that “lexical cognates are all but non-existent” [29: 204]. Again, one may suspect that both the original classification by Laycock and Z’graggen and its elaboration by Foley have a geographic bias and are different, in this respect, only in terms of geographic granularity.

Only in the last few decades has the correspondence between the geographic dispersal of languages and their linguistic divergence become a research question rather than a potential for an implicit bias in language classification. One of the first models explaining geographic distribution of linguistic features was Trudgill’s [30] Gravity Theory (cf. also [31]). This model implied that linguistic innovations do not evenly spread over a given dialect continuum. Instead, they spread via the most influential centers, from which they move to smaller ones, with the latter being the source of innovation for even smaller centers, thus creating a “cascade effect”. Although this model was criticized afterwards, it gave a strong impulse to the development of theories and models of geographic dispersal of languages and linguistic features. The criticism of the model was based on empirical testing: in [32] Nerbonne, van Gemert and Heeringa show that “geography indeed plays an overwhelming role, […] there is no dominant gravity-like (inverse-square) force evident in the residue of linguistic differences, and […] the role of population, while weak, is actually the opposite of that postulated by the gravity model.” See also [33], where praise and criticism of the Gravity Theory are discussed in detail.

Subsequent research has focused on several directions.

*Geolinguistics* primarily attempted to trace the histories of individual features and collections of features [34]. The analysis of geographic diffusion in geolinguistics incorporated multiple characteristics of variationist sociolinguistics [35,36] as well as environmental factors in the diffusion of features (cf. [37–39]). A range of studies in geolinguistics focus specifically on the features that do not map well to geographic distributions in an attempt to understand the relations between geographic and linguistic divergence of language varieties (cf. [40]) and convergence of typological profiles, often at a macro-level [41].

*Dialectometry* adopted spatially oriented approaches similar to the wave theory (Wellentheorie) and Gravity Theory in the study of language change and enriched them with a quantitative perspective (cf. [42–46]). As compared to geolinguists, dialectometrists attempt to infer spatial patterns of variation from large datasets based on aggregated differences rather than from analysis of individual features (cf. [47]). Dialectometry works at a high level of spatial granularity and deals with closely related linguistic varieties. This approach is essentially non-phylogenetic due to the limitations of cladistic representation in its application to dialect continua [48]. It does not rely on modeling linguistic innovation and retention and does not translate into phylogenies. It uses its own similarity measurements, such as aggregated phonetic differences, and relies on other types of representation adapted to reflect continuous variation, such as heatmaps and multidimensional scaling.

Finally, advances in computational modeling opened new perspectives for *phylogenetic research* in linguistics (cf. [49–52]). As developments in traditional comparative linguistics allowed to link the spread of lexical items to the spread of cultural features (e.g. animal husbandry), material objects (such as, e.g. crops or ceramics) and human groups (cf. [14,53,54]), the plausibility of particular reconstruction hypotheses significantly increased or decreased. Larger amounts of extralinguistic evidence can now be taken into account to calibrate more conventional (e.g. relying on tree-like representations [49–52]) and justify novel (e.g. using networks [55]) computational methods in comparative linguistics. This research essentially focuses on large-scale genealogical relations between languages, such as topologies of whole linguistic families (at this scale of comparison, a family the size of Dravidian may be considered a *small genealogical unit*, cf. [50]). How strong the correlation between geography and language divergence is expected to be is rarely asked, with a major proportion of research focusing on explaining linguistic diversity (or lack thereof) across various geographic areas and landscapes (cf. [56–59]).

In this paper, we attempt to bring these three lines of study together by using the phylogenetic approach and its patristic distances at a geographic scale comparable to that of dialectometry, and checking their goodness of fit with geographic distances, one of the central research questions within geolinguistics. We use data with high geographic granularity from an area known for its language density to map language distributions onto geography. We consider 77 villages, in which 8 languages that compose the Andic branch of the East Caucasian language family are spoken. These villages lie in a small foothill-to-highland area in the northern Caucasus (Daghestan, Russia), comprised within one square degree (northern latitude 42.1º to 42.9º, eastern longitude 45.7º to 46.6º, roughly 6,600 km^2^, which is about nine times smaller than the span of the dialects of Dutch). We compare phylogenetic and geographic distances between the languages whose divergence is comparable to that of the Germanic branch of Indo-European (see [60,61] for lexical comparison; and Alekseev [62], Mudrak [63: 4] for different time depth estimates). We intentionally focus on a small area with great detail in locations, complex landscape and several reconstruction hypotheses proposed in various studies [61,63–67] in order to check the applicability of a phylogenetically oriented analysis at a small scale, with several competing classifications included in the comparison. We apply Congruence Among Distance Matrices (CADM [68,69]) measure to test the hypothesis of whether, even at such a small geographic scale, geographic distances correlate with linguistic phylogeny.

The aims of this paper are, first to test the correlation between phylogeny and geography and, second, to investigate how this correlation depends on the specific methods used for building the phylogeny. In particular, we test how quantitative and qualitative phylogenies are compared in this respect.

The results of the study are as follows. For all phylogenies suggested in the literature on Andic, we find that the correlation with geographic distances is above random, indicating that geographic distance is, in this case, a viable predictor of linguistic differentiation. We also observe that the classifications based on considerations other than lexical comparison [64,65,67] show a better fit with the geography than classifications based on cognacy of Swadesh list items [66,61]. We interpret this difference as a consequence of the fact that traditional qualitative classifications have an implicit geographic bias or are based on selections of isoglosses that naturally tend to behave better in spatial terms than shared lexical retentions and innovations, on which quantitative phylogenies are usually based.

We conclude by saying that the observed distributions of languages—and therefore the actual geographic distances between them—are a consequence of complex historical processes such as human migrations and language shift, for which the models we use in this study do not account. As an outlook of the study, we suggest that lexicon-based models may be refined with priors accounting for the competing views on the dynamics of language spread, thus yielding models with the same linguistic module but different historical priors. These models may in turn be meaningfully compared to each other as to how they account for the present-day geographic distribution of languages.

The remainder of the paper is organized as follows. In Section 2, we describe the classifications proposed for Andic languages, and their controversies. In Section 3, we explain the materials and methods used. In Section 4, we describe our results. In Section 5, we discuss the results obtained, and in Section 6, we enumerate our conclusions.

## 2. Classifications of Andic languages

Andic languages form a branch of the East Caucasian (also known as Nakh-Daghestanian) language family. This branch is traditionally divided into eight languages, namely Akhvakh, Andi, Bagvalal, Tindi, Godoberi, Chamalal, Karata, and Botlikh (see e.g. andi1254 in Glottolog [70]). One of the reasons for choosing this particular language group for our study is that several more or less conflicting phylogenetic hypotheses have been put forth, which may be compared in terms of correlation with geography.

**Fig 1:**
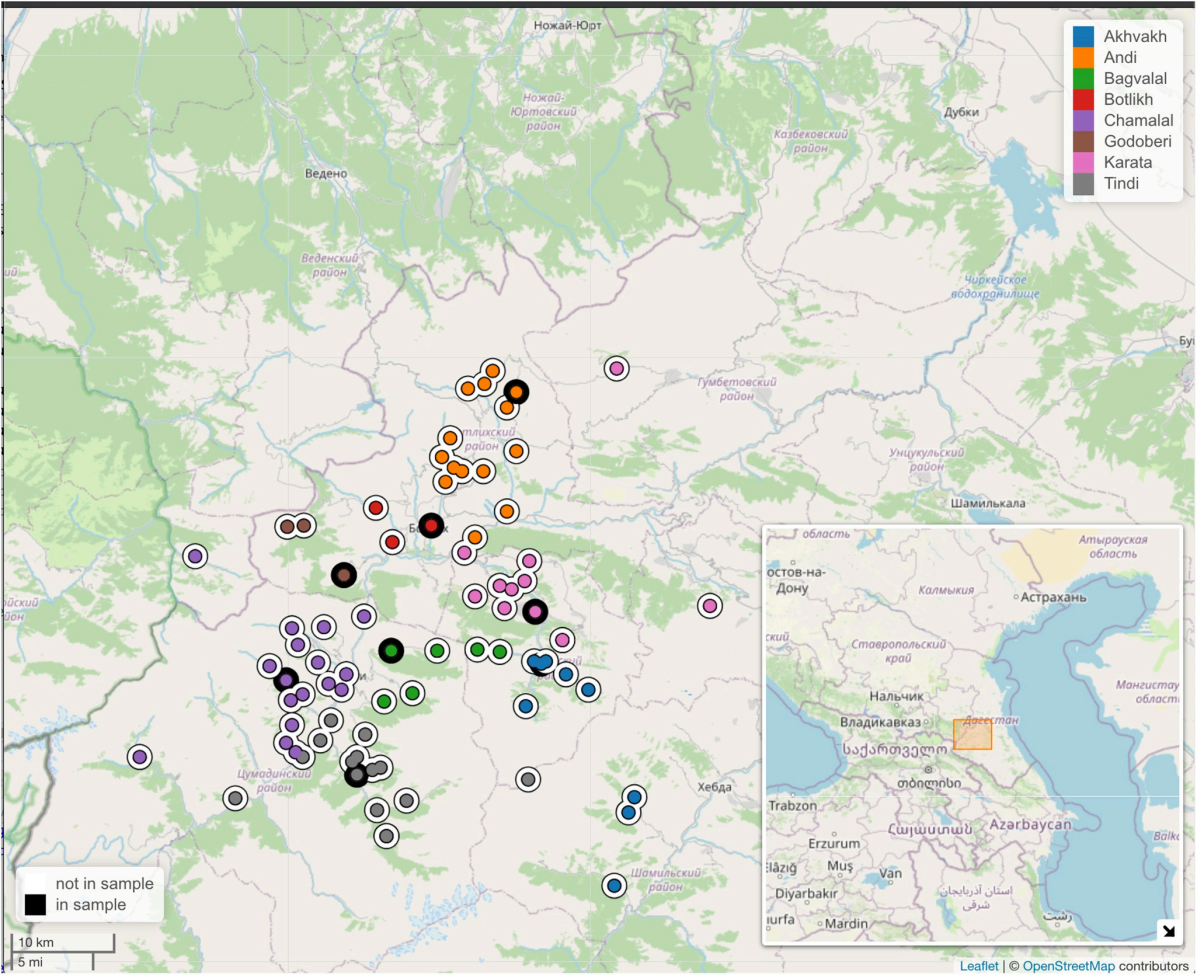
Villages considered in this study. Each color corresponds to one language, and dots surrounded by a black ring indicate the villages used as representatives of each language. Base map and data from OpenStreetMap and OpenStreetMap Foundation.

Fig 1 presents our region of interest. It shows all 77 villages in which the varieties of the 8 Andic languages are traditionally spoken (excluding recent re-settlements) [71]. All graphs and maps in this paper were created with the *R* programming language [72] using the following packages: *ggplot2* [73] and *lingtypology* [74].

Classification of Andic languages is complicated for two main reasons. First, it has been suggested that Andic languages as a whole may in fact represent a continuum without clearcut language boundaries (cf. [75:272], repeated in [65]). Second, some village varieties of what is traditionally considered one language may be highly divergent from its other varieties, arguably constituting separate languages (this applies to the Chamalal spoken in Gigatli; the Karata spoken in Tukita; Lower Andi dialects as compared to Upper Andi dialects; and South Akhvakh dialects as compared to North Akhvakh dialects).

**Fig 2:**
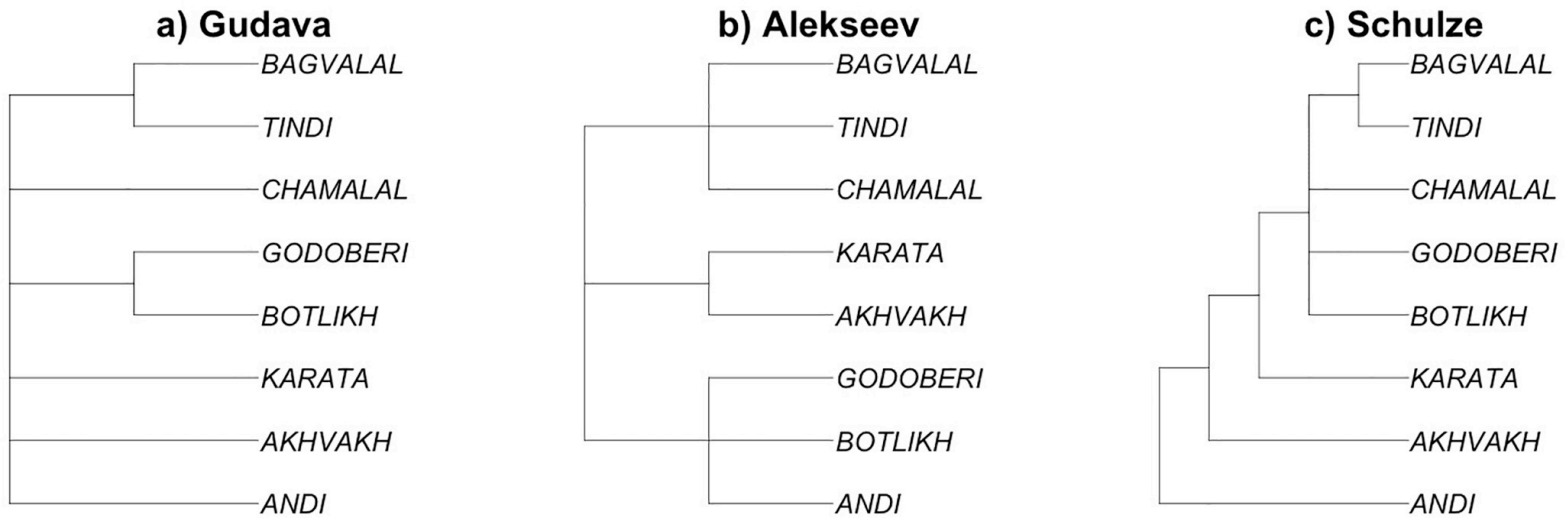
Qualitative approaches to the classifications of Andic languages. Tree tips represent languages, and branch lengths are all plotted equally.

Several classifications of Andic have been suggested starting from the 1950s, with criteria often anything but explicit (Fig 2). Gudava, in his *Comparative analysis of verbal stems in the Avar and Andic languages* [64:3–4], see also [75:272] (Fig 2a), essentially represents the Andic languages as a flat structure except for classifying Godoberi and Botlikh, on the one hand, and Tindi and Bagvalal, on the other, as dialects rather than languages, while they are classified as different languages in subsequent literature. His data include both his own fieldwork and previous descriptions [76,77] as well as unpublished fieldnotes (e.g. Magomedbekova’s data on Akhvakh and Karata). In [65] (Fig 2b), Alekseev suggests three branches—Andi-Botlikh-Godoberi, Karata-Akhvakh, and Bagvalal-Tindi-Chamalal—on the basis of what he calls his “observations” [65:3]. In a sense, his whole book on comparative morphology, from which this classification is extracted, is a collection of such comparative observations; and the same is true of Gudava’s work [64]. In an online manuscript, Schulze [67] (Fig 2c) suggests the most sophisticated qualitative tree of all, splitting off first Andi, then Akhvakh, then Karata, and then dividing the rest into four groups, including three separate groups for Botlikh, Godoberi, and Chamalal, and Tindi and Bagvalal together in the fourth one. This short unpublished manuscript only contains the tree but no linguistic arguments substantiating it. None of these three qualitative classifications seems to be based primarily on lexical divergence, and no methodology is provided. When using them, all one can rely upon is the expertise and the authority of their authors, who may cross-reference each other but in the end provide neither isomorphic classifications, nor explicit criteria of linguistic (dis)similarities to falsify their suggestions.

Lexicon-based classifications of Andic have been only recently suggested, including those based on Swadesh lists by Koryakov in [66] and Filatov & Daniel in [61], and on a wider selection of lexicon by Mudrak in [63]. In the first two studies, most data come from field elicitations, but a few word lists are from published sources, including [78,79] and Sergei Starostin’s comparative database [60]. Koryakov uses the StarlingNJ method as a built-in function of the Starling database (see [60,80] for a brief description of StralingNJ). Filatov and Daniel make a phylogenetic reconstruction based on Markov Chain Monte-Carlo using BEAST2 [81] (note that [61] is a database which is constantly updated by data from new villages so the topology of the tree may be changing). Sources used by Filatov & Daniel and Koryakov, and even the varieties included in their studies (villages of the provenance of the lexical data) only marginally overlap. Given that village varieties of the same Andic language may be strongly divergent, the two lexicon-based phylogenies may be considered by and large independent. Mudrak, on the other hand, is not explicit about his computational methods, but it is very likely that he uses the same Starling NJ method as in Koryakov [66]. His tree [63: 8] is based on cognacy annotations of more than 3,000 lexical items. As to the varieties included in his study, for some languages he does not provide an explicit indication of the dialect or village the data come from. For the sake of comparison below, we are assuming we can use the villages that are indicated as the main sources of lexical data in the dictionaries he says he is using [63: 9–11]).

**Fig 3:**
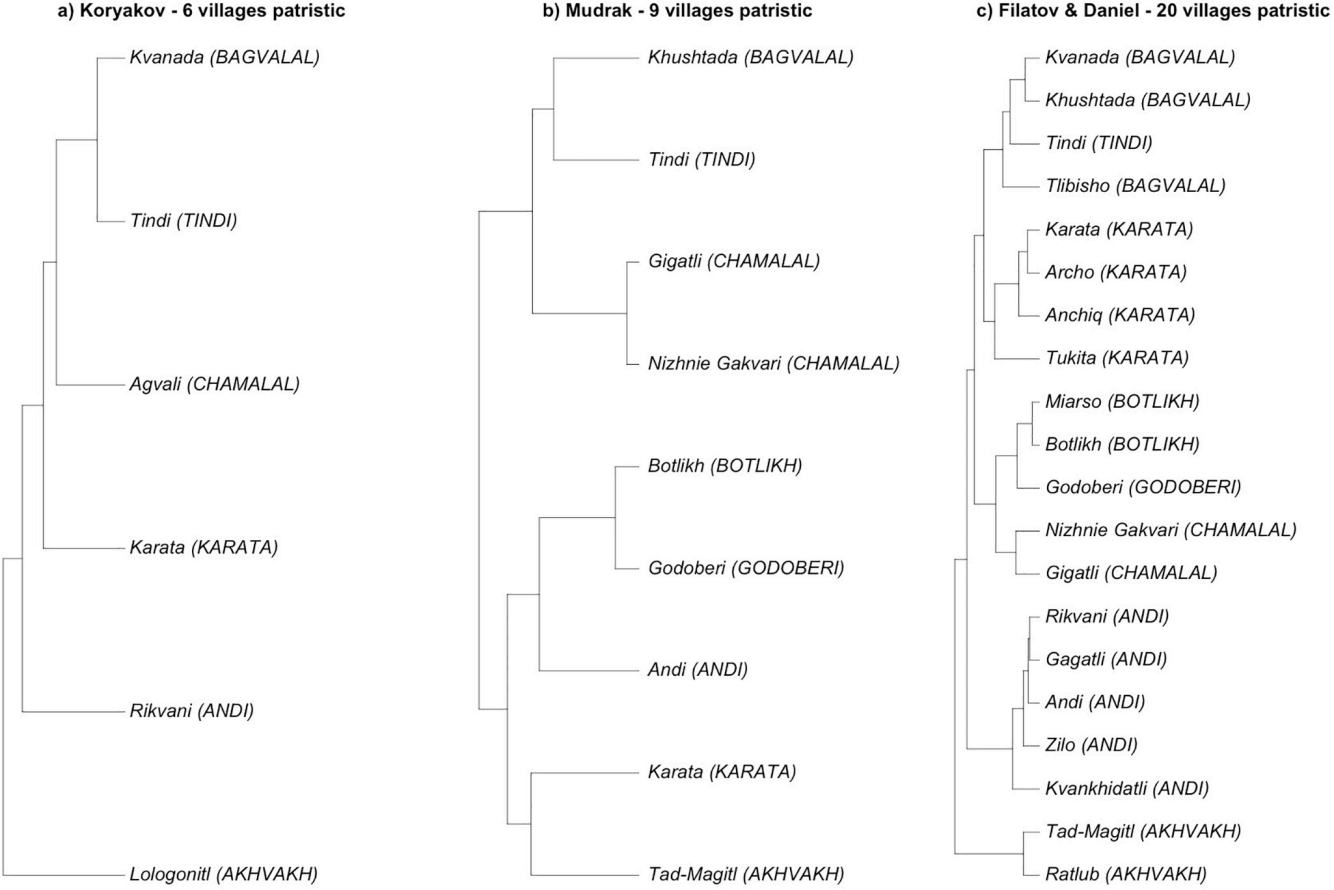
Quantitative lexicon-based phylogenies for Andic languages. Tree tips represent villages rather than languages, and branch lengths are a meaningful measure of the phylogenetic distance between languages. For the tree in (b), the villages are imputed according to the “main dialect” indicated in the sources used by Mudrak [63].

The full trees by Koryakov [66], by Mudrak [63], and by Filatov & Daniel [61] are shown on Fig 3. Koryakov and Filatov & Daniel’s trees deliver similar topologies. Bagvalal (represented by Khushtada, Kvanada and Tlibisho in Filatov & Daniel and by Kvanada in Koryakov) is classified together with Tindi; and Akhvakh (represented by Tad-Magitl and Ratlub in Filatov & Daniel, and by Lologonitl in Koryakov) remains an outlier. Note that Fig 3a only shows the languages for which Koryakov carried out lexical analysis; on the actual tree he provides, he also shows Botlikh and Godoberi whose positions he imputed based on qualitative considerations, adding them to the same node as Chamalal and Bagvalal and Tindi (as in Schulze [67]). On the other hand, Mudrak’s tree is very similar to that suggested by Alekseev (see Fig 2b, where Akhvakh merges with Karata, while Andi merges with Botlikh and Godoberi), whereas the classifications obtained by both Koryakov and Filatov & Daniel converge at making Akhvakh and then Andi early splitters (in this order).

**Fig 4:**
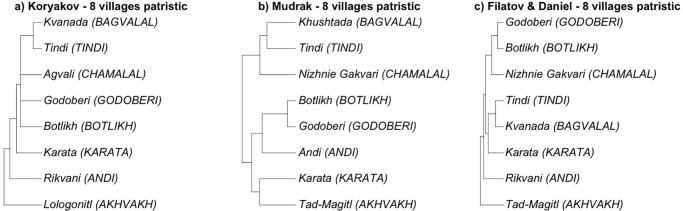
Quantitative lexicon-based phylogenies for Andic languages. Tree tips represent villages rather than languages, and branch lengths are a meaningful measure of the phylogenetic distance between languages. For each tree, eight villages are included (compare with Fig 3).

Filatov and Daniel’s tree (Fig 3c) is more granular than thoseby Koryakov (Fig 3a) and Mudrak (Fig 3b). It includes some lects that are conventionally considered the same language, and are indeed linguistically close in lexicon-based phylogenies besides being also close neighbors in terms of geography. Examples of this are the lects of Rikvani, Gagatli, Andi and Zilo for the Andi language (corresponding to Rikvani in Koryakov’s tree); the lects of Miarso and Botlikh for the Botlikh language; the lects of Kvanada and Khushtada for the Bagvalal language (corresponding to Kvanada in Koryakov’s tree); and the lects of Karata, Archo and Anchik for the Karata language (corresponding to Karata in Koryakov’s tree)—see S1 Table in S1 Data for the full list of villages and languages. Including more geographically and linguistically close varieties may obviously boost the correlation between phylogenetics and geography (cf. Fig 6 and discussion below). Filatov and Daniel’s tree also includes some highly divergent lects, such as Gigatli for the Chamalal language and Anchik and especially Tukita for the Karata language; their impact on a comparison with a phylogeny that does not include them is hard to predict. In order to make it possible to meaningfully compare the phylogenies, we had to reduce the number of lects in Filatov and Daniel’s tree so as to match the other trees. We did so by subsampling the same village lects as used by Koryakov (Rikvani for Andi, Kvanada for Bagvalal, Karata for Karata), or substituting them with presumably closest matches in cases where Filatov and Daniel did not have data from the same village (matching Tad-Magitl and Lologonitl for the Akhvakh language; Nizhnie Gakvari and Agvali for the Chamalal language); this also excluded the divergent lects. The two resulting phylogenies are shown in Fig 4, with Fig 4a showing Koryakov’s phylogeny including the imputed Botlikh and Godoberi; Fig 4b showing a version of Mudrak’s phylogeny excluding the Gigatli variety of Chamalal; and Fig 4c showing a reduced version of Filatov and Daniel’s phylogeny that matches the village lects used by Koryakov as closely as possible.

**Fig 5:**
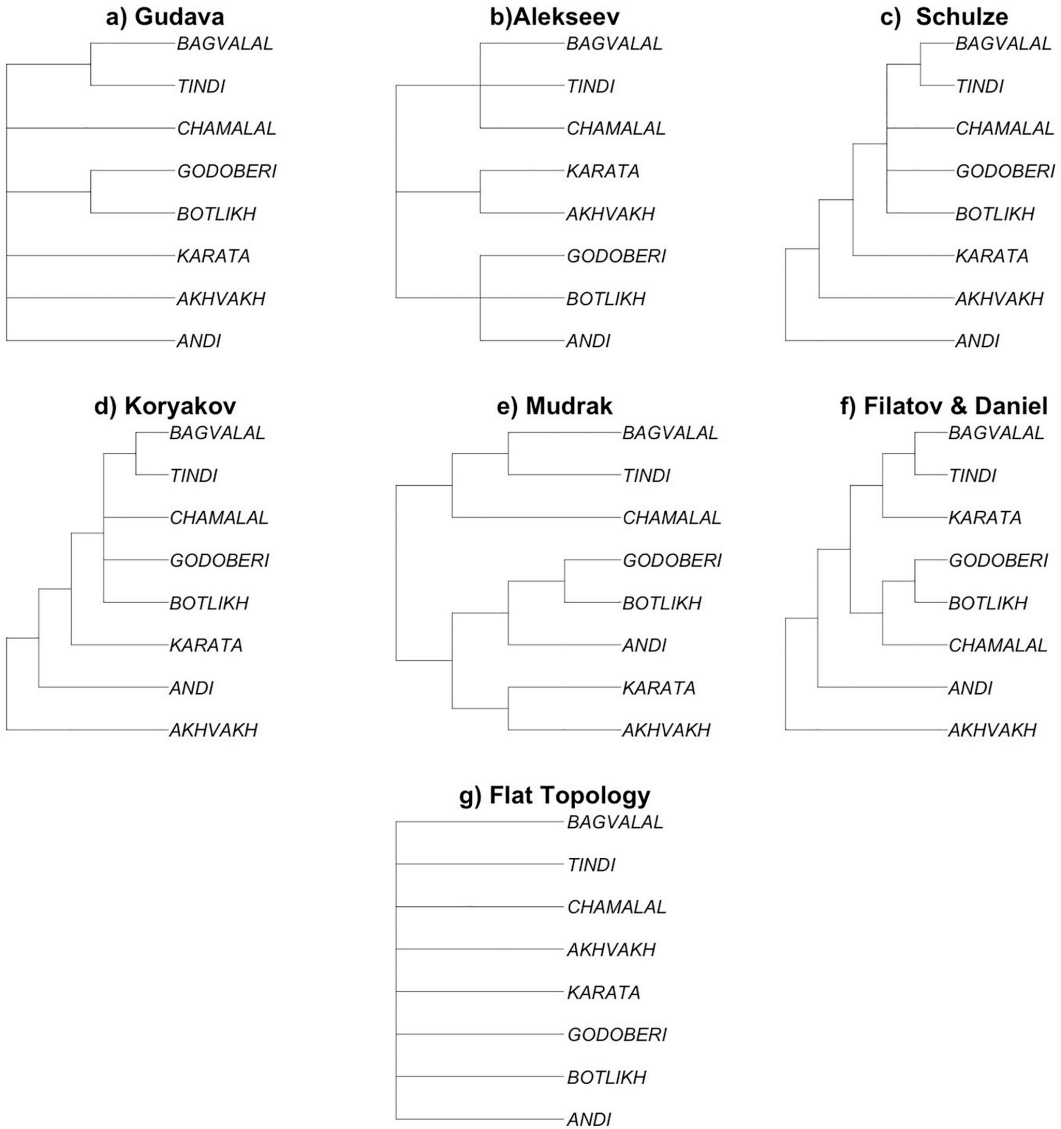
Qualitative classification and lexical phylogenies aligned for comparison. Tree tips are languages, and branch lengths are all plotted equally. Trees are ordered according to the time of the publishing. Flat Topology was added as a baseline for comparison.

Another issue with comparability of phylogenies is that, unlike the qualitative trees shown in Fig 2, the branch lengths of the trees on Fig 3 and Fig 4 are meaningful, proportional to the linguistic and/or chronological separation between lects. In order to make the comparison between qualitative and quantitative trees possible, we need to treat quantitative phylogenies as qualitative ones; in other words, to consider the branch lengths in the phylogenies in Fig 4 not meaningful. As a result, we obtained all classifications aligned in terms of eight languages, as shown in Fig 5. We also added a “flat topology”, agnostic to any internal structure of Andic languages, that will be used as a baseline for comparison in the following sections.

In what follows, we investigate which of the classifications in Fig 5 show a better fit with geographic distances, and compare the strength of this association. Phylogenetic distances for these classifications are calculated node-wise, as a number of the nodes between the two tips of the tree. Correlations with the quantitative phylogenies in Figs 3 and 4 are provided for comparison.

## 3. Materials and methods

### Linguistic data and distances

The data for the languages spoken in each village of the dataset were taken from the East Caucasian villages dataset [71]. Detailed information about the dataset is available at the GitHub page of the project (see the Data Availability Statement for the link). The phylogenetic trees used for calculating phylogenetic distances are based on [61,63–67], as discussed in Section 2. Linguistic distances were calculated from the different classifications in Figs 3–5, with the *ape* R library [82]. We calculated the distances as the number of nodes between the location of two languages in a tree for all phylogenies including [61,64–67], as shown in Fig 5; but also the original patristic distances in case of Mudrak’s [63], Koryakov’s [66] and Filatov & Daniel’s [61] as shown on Fig 3, and the 8-languages patristic distances for the same trees, Fig 4, for comparison. For the sake of comparison, a flat topology was added, where all languages are equally related (Fig 5g).

### Geographic data and distances

We calculated two types of distances: i) *great circle distances* (GCD), i.e. the shortest way between villages over a spherical surface, and ii) *travel cost distances*, which take into account the landscape, a potentially important factor in language divergence in highland Daghestan. Landscape data come from the Shuttle Radar Topography Mission (SRTM [83]). In calculating travel costs, granularity of 90m × 90m areas per pixel was used, since the results were not sensibly different when using 30m × 30m instead. We calculated the travel cost for all pairs in our database of 77 villages. Travel costs were calculated from slopes and transversal cost with the functions *create_slope_cs* and *create_transversal_cs* with 16 neighbors from the R library *leastcostpath* [84]. The former creates a cost surface based on the difficulty of moving up/down slope, by using Tobler’s ‘Hiking Function’, while the latter adds the difficulty of traversing across slopes with Bell and Lock’s algorithm [85]. However, travel cost differences showed a very high correlation with GCD (see S1 File in S1 Data). As a result, in what follows we use simpler GCD-based models instead of more complex travel cost-based ones. Calculations using travel cost distances are reported in S10-S12 Figs in S1 Data.

### Comparisons

Comparisons between linguistic and geographic distances with the Congruence Among Distance Matrices (CADM) algorithm [68,69] were performed using the *vegan* library [86] for R. A significance check was performed by recalculating these correlations after randomly shuffling the languages in each phylogeny (see Results).

### Analysis

Our analysis was intended to investigate how the classifications of the Andic languages discussed in Section 2 correlate with geographic distances. In the first experiment, we used patristic distances as shown in Figs 3 and 4 for the topologies available, and node-wise distances in the qualitative classifications shown in Fig 2 (including phylogenetic classifications from adapted by discarding information on branch length, as in Fig 5).

In the first experiment, correlations between linguistic and geographic distances were calculated for 8 villages, each one representing one language. The sources of qualitative classifications do not indicate the specific villages whose lects they classify (cf. Fig 2). For these classifications, one village representative of each language was chosen. The selection of villages was the same as in the subsample of the villages of Filatov and Daniel’s dataset (namely, Rikvani, Botlikh, Godoberi, Karata, Kvanada, Tindi, Nizhnie Gakvari, and Tad-Magitl’ representing the languages Andi, Botlikh, Godoberi, Karata, Bagvalal, Tindi, Chamalal, and Akhvakh, respectively).

In the second experiment, we addressed the problem of having the same language spoken in different villages. This creates pairs of varieties with the same linguistic distance (e.g. languages A and B), but different geographic distances (e.g. villages A1 vs B1 and A2 vs B2). We tested the impact of this issue on our results by carrying out a permutation test on the whole set of villages, as explained below.

We perform pairwise comparisons of the linguistic phylogenies against the geographic distances with the method of Congruence Among Distance Matrices (CADM, [68,69]). This is an extension of the Mantel test of matrix correspondence, used to test the null hypothesis of complete incongruence of the distance matrices. Given two or more datasets (in this case, trees) studied on the same species, a concordance statistic known as Kendall’s W [87,88] is calculated among the distance matrices corresponding to the trees, and tested against a distribution of permuted values to estimate the probability that the data correspond to the null hypothesis. The W statistic gives an estimate for the degree of congruence of the matrices on a scale between 0 (null hypothesis, no congruence) and 1 (complete congruence) [68].

To make all phylogenies comparable, phylogenetic distances between languages were calculated based on topologies in Fig 5, taking the amount of nodes between two languages as linguistic distance between them. All code is available in S3-S7 File in S1 Data, as well as in the OSF repository (see Data Availability Statement).

## 4. Results

**Fig 6:**
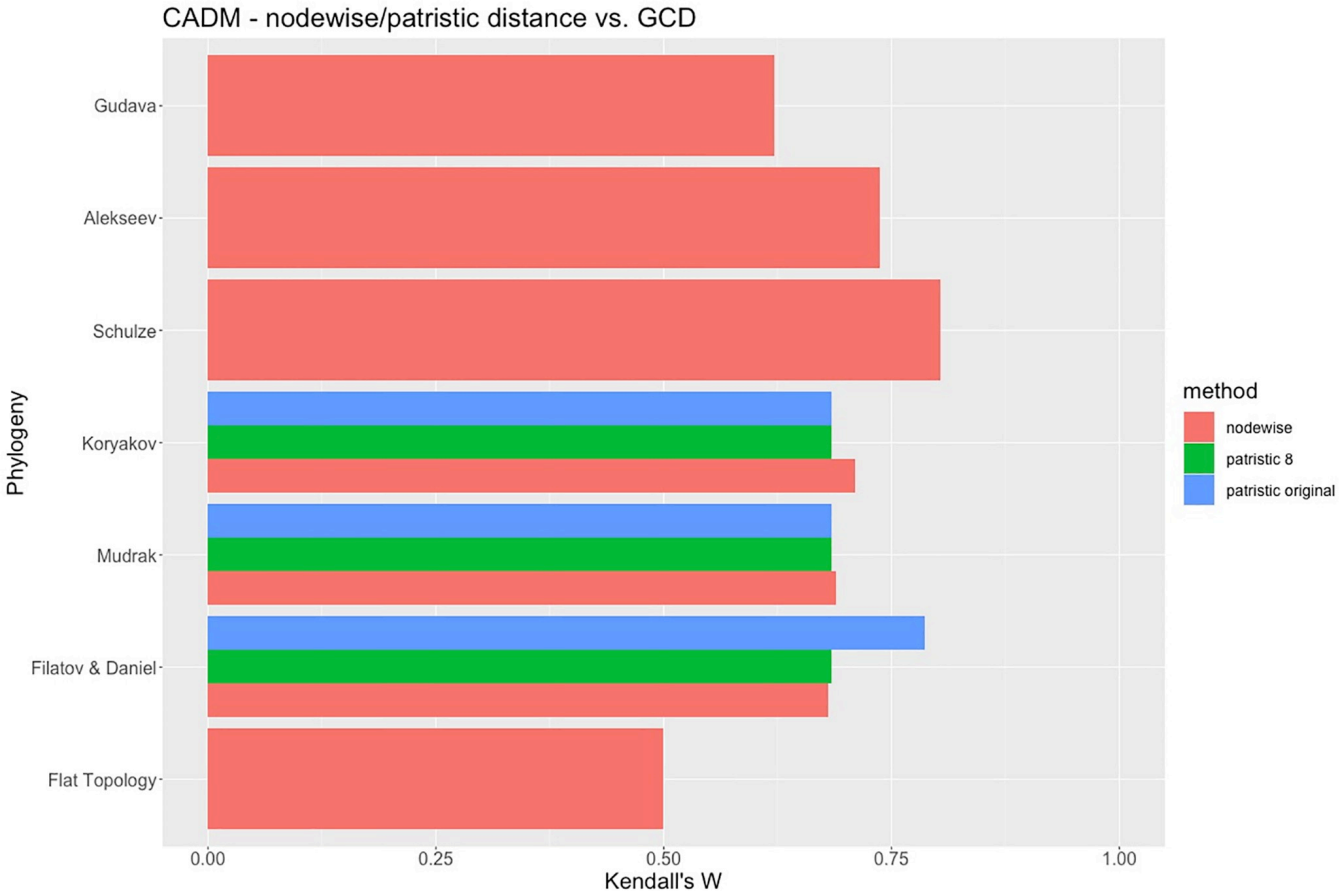
Kendall’s W for the congruence of each phylogeny with geography. Red bars consider nodewise normalized phylogenies, for 8 villages. Green bars consider patristic distances for quantitative phylogenies, restricted to 8 villages. Blue bars consider patristic distances for quantitative phylogenies, using the original data in each case (6 villages for Koryakov, 9 for Mudrak, and 20 for Filatov & Daniel).

Fig 6 provides a comparison of the correlation of each of the classifications with geographic distances, shown as the value of Kendall’s W from CADM.

Red bars use node-wise distances and are available for all phylogenies. For comparison, we also provide correlations obtained for patristic distances. Blue bars use patristic distances as represented in Fig 3, only available for Koryakov [66], Mudrak [63], and Filatov & Daniel [61]. Green bars use patristic distances as represented in Fig 4 (upgraded from 6 to 8 villages in the case of Koryakov, reduced from 9 to 8 in the case of Mudrak, and from 20 to 8 in the case of Filatov and Daniel).

All classifications outperform the baseline classification (“flat typology”), in which all linguistic distances are the same (and W necessarily equals 0.5). Schulze’s [67] classification has the higher correlation, closely followed by Alekseev in [65]. In the case of Gudava [64], the correlation with the geography is the lowest of all. We suggest that this is due to the fact that his topology is almost flat, with only two sub-branches (see Fig 2a), which in fact he considers to be dialects. In a sense, this is not a phylogeny but grouping of varieties into languages. As a result, the correlation with the geographic distances in the case of this quasi-flat topology is only better than the baseline, truly flat topology with W = 0.5 (Fig 5g).

Koryakov’s lexicon-based phylogeny [66], for which we also have a phylogeny with meaningful branch lengths (green and blue bars), performs in roughly the same way whether we use patristic distances or calculate the distances nodewise (cf. the red bar). The difference between the blue bar (the original six villages for which Koryakov used lexical data) and the green bar (with the position of two additional languages imputed) is all but non-existent. The same is true for Mudrak [63]. In the case of Filatov & Daniel’s lexicon-based phylogeny [61], patristic distances for eight villages (green bar) perform as good as node-wise distances (red bar), and as Koryakov’s and Mudrak’s phylogenies. For all the 20 village varieties (blue bar), instead, the correlation is much higher than for most other phylogenies, only slightly lower than Schulze’s. As we suggested in Section 3, this is due to the fact that this classification includes many more locations with close varieties, conventionally considered the same language, which strongly boosts the correlation between this phylogeny and geographic distances.

For the sake of comparability, let us only consider the values shown as red bars. Essentially, the three lexicon-based phylogenies perform the worst in terms of correlation with geographic distances. The only qualitative phylogeny that performs worse than these three is Gudava’s quasi-flat topology, whose low correlation directly follows from the almost complete absence of structure in his tree. As we discussed, the flatter the topology is, the closer is the correlation to W = 0.5. More generally, a certain level of correlation may be innate to the topology of the tree.

**Fig 7:**
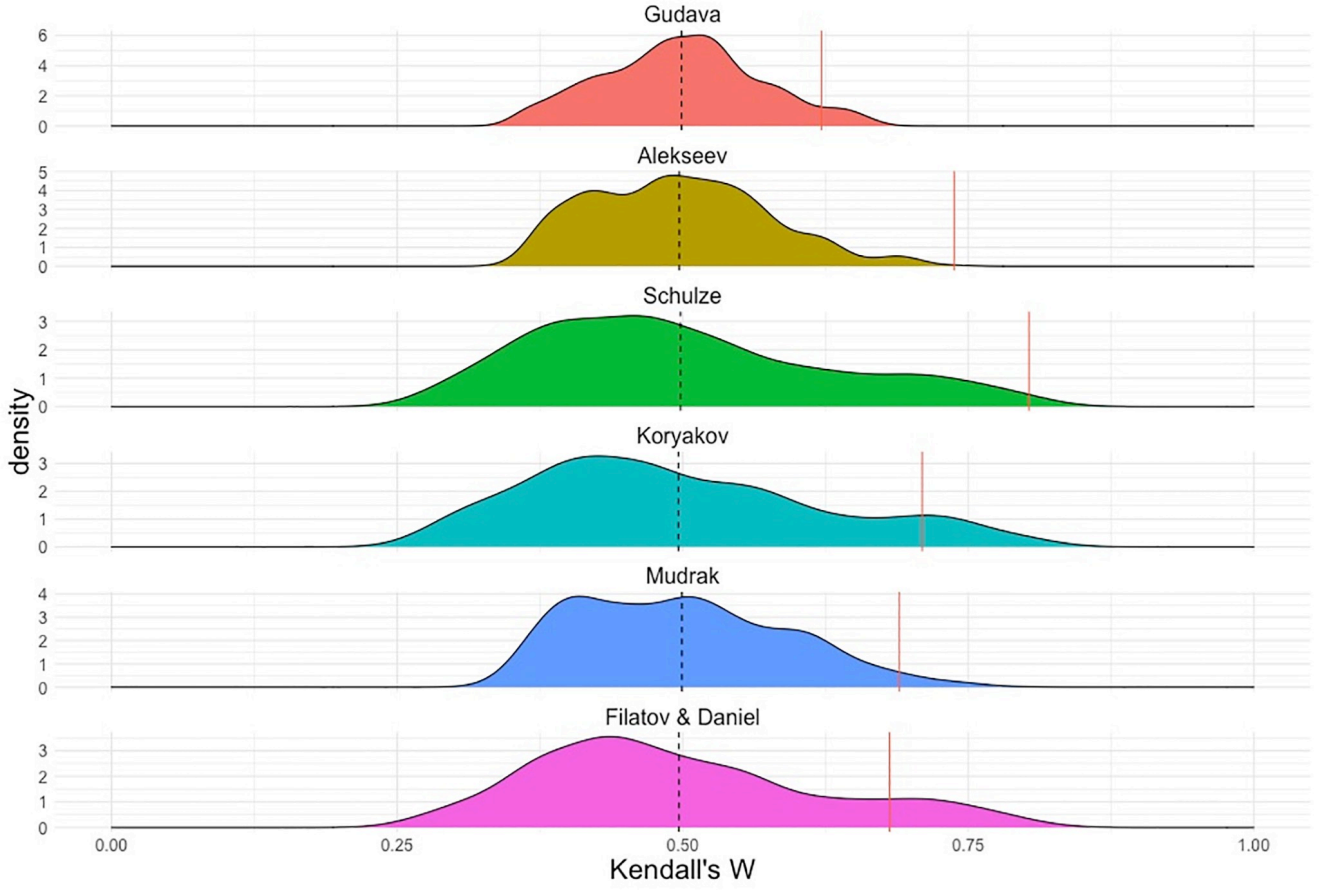
Distributions of Kendall’s W for permuted phylogenies (1,000 permutations). Dashed lines represent the mean of the distribution (W = 0.5 in all cases), andfull red lines represent the observed value for the phylogeny, as red bars in Fig 6.

To control for the significance of the correlation level in each tree, we ran a permutation test in the following way. For each phylogeny in Fig 5, we randomly shuffled the tips’ names (eight languages) and then calculated Kendall’s W for the resulting tree. In order to test for significance, we define a statistic as how often, out of the 1,000 permutations, a permuted (i.e. random) phylogeny performs better than the actual one. The results are shown in Fig 7.

The values of the statistic are 0 for Alekseev, 0.007 (i.e. 7 out of 1,000) for Schulze, 0.026 for Mudrak, 0.046for Gudava, 0.088 for Koryakov, and as high as 0.116 for Filatov & Daniel. Notably, the classifications based on lexical divergence tend to show higher values (weaker significance) while those based on qualitative considerations tend to show lower values (stronger significance). These results suggest that, *given their respective topologies*, lexicon-based phylogenies are not the ones most aligned with the geography, while the qualitative ones, again *given their topologies*, are statistically among the most well-aligned. Since lexicon-based phylogenies assumedly cannot include a geographic bias and are based solely on lexical retentions and innovations of basic vocabulary, which is usually considered a more reliable tool at this time depth, they can be taken as a gold standard (see [41:2] on the time depth of various reconstruction tools). With this in mind, the numbers yielded by the test suggest a geographic bias in the qualitative phylogenies.

**Fig 8:**
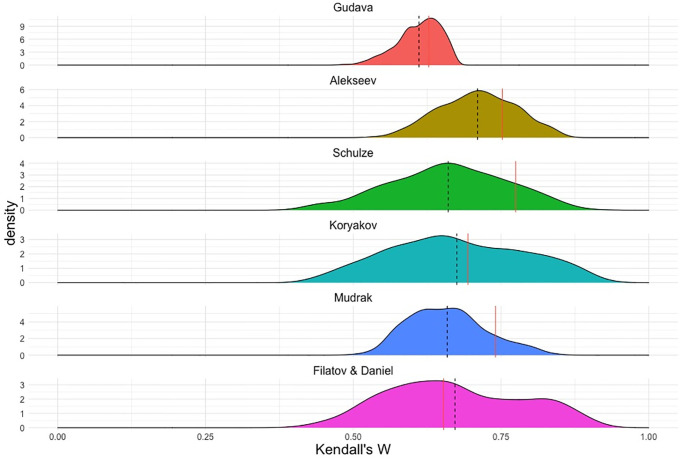
Distributions of Kendall’s W for re-sampled village sets (1,000 permutations). Dashed lines represent the mean of the distribution, andfull red lines represent the observed value for the phylogeny, as red bars in Fig 6.

As discussed above, for the qualitative classifications in Fig 2, the languages were mapped to the set of representative villages for each language, which is essentially an *ad hoc* selection for all phylogenies and might lead to a sampling bias in calculating a correlation. This bias can be controlled for by randomly sampling one representative village per language, and re-calculating Kendall’s W. In this sampling we use the locations of all 77 villages in Fig 1, and not only the 8 pre-selected ones. The result is shown in Fig 8, where 1,000 random permutations were performed.

In the random village sampling, all classifications continue to outperform the baseline of flat typology, W = 0.5. We interpret this as an indication that the correlation with geographical distances we observe in the phylogenies is not due to a sampling bias in the selection of the eight representative villages. There is also an effect of the internal topology of the classification in the sense that e.g. Gudava’s quasi-flat topology shows a smaller dispersion. This is not a meaningful difference between classifications but just a technical consequence of the fact that, in a flatter topology, the difference between the highest and the lowest value of the distance (the number of nodes) between two languages is lower than in more complex topologies with a higher amount of branching (consider also Alexeev’s phylogeny as a somewhat intermediate case). Apart from this, the distribution for Alekseev’s classification is displaced to the right, which means that, on the average, it shows a better correlation with geography than other classifications, though others such as Filatov & Daniel’s [61] and Koryakov’s [66] but also Schulze’s [67] can marginally show a higher correlation because of the higher dispersion, depending on the villages chosen.

As one statistic for testing this, we can look at the percentage of the permutations that, for a given phylogeny, deliver the value of Kendall’s W below W = 0.5, the baseline distribution. This gives us an idea as to whether a phylogeny correlates with geographic distribution of the languages in general, regardless of what villages we choose to calculate distances between languages. The values for this, after 1,000 permutations, are: 0 for Alekseev, 0.001 for Mudrak, 0.004 for Gudava, 0.05 for Filatov & Daniel, 0.056 for Schulze, and 0.064 for Koryakov. In this sense again, the phylogenies based on lexical divergence, the most conventional way of calculating linguistic divergence, perform the worst, although still hovering around the threshold of the standard 0.05 significance level. The fact that this time they are joined by Schulze’s topology suggests that this, too, may be a consequence of the tree complexity. Indeed, sampling different villages when the languages corresponding to them have a zero node-wise distance does not affect the correlation between phylogeny and geography. In a flatter topology, there are more languages with zero distances node-wise (such as Akhvakh and Karata for Gudava and Alekseev, but not for the other classifications). As a result, flatter topologies are more, and complex topologies are less immune to village sampling.

In a slightly different perspective, we can look, for specific phylogenies, at the position of the observed value of Kendall’s W with respect to the median of the distribution. All phylogenies except Schulze’s show mean values that are relatively close to the observed values. And even in the case of Schulze’s phylogeny, the difference is not significant. We can conclude that the samples we are using are representative in terms of geographic sampling.

In general we conclude that the relation between the linguistic phylogeny and the geographic distances is, in our case, relatively immune to how the specific locations for languages are chosen (i.e. to village sampling).

Another possible way for dealing with this bias would be to generate a larger tree, where we include all 77 villages, placing all the villages with the same language in the same place of the phylogenetic tree (imputed tree). This was implemented, but the differences among topologies became practically imperceptible because of the amount of shared assumptions about the internal topology of dialects for each language. This is discussed in more detail in S2 File and S2-S8 Figs in S1 Data.

## 5. Discussion and future research lines

From the analysis above, we can clearly see that most phylogenies proposed for the Andic languages correlate with geographic distances better than chance (Fig 7). However, the trees that are based on qualitative (and not always explicit) criteria, such as Alekseev and Schulze’s trees [65,67], show a higher correlation with the geography than those generated using lexically oriented phylogenetic methods based on common innovations. Though the validity of the specific results presented here is limited to the Andic data, the proposed method of comparison itself can be extended to other language groups.

Our interpretation is that when building qualitative classifications, researchers may naturally bend towards geographically meaningful isoglosses. Alekseev himself seems to admit a certain degree of circularity in his classification; cf. (translation is ours):

“According to our observations, it is possible to isolate three subgroups of Andic languages, including Andi-Botlikh-Godoberi, Karata-Akhvakh and Bagvalal-Tindi-Chamalal. It is noteworthy that this classification corresponds to the different geographic sub-zones where the Andic languages are spoken, including the Botlikh district, the Akhvakh district and the Tsumada district. In its turn, apparently, it could not but entail a certain degree of areal bias in the selection of some of the classificatory features”

[65:3].

This is coupled with important differences in the topology of his tree, as compared to other topologies, that may follow from his geographic bias. He classifies Akhvakh together with Karata, and Andi together with Botlikh, which does seem to be strongly influenced by the location (if not administrative district affiliation!) of the villages. In other classifications, both Akhvakh and Andi appear as outliers (even if in different orders, depending on classification; cf. Fig 5). This example illustrates that, while the use of geographically-oriented linguistic data such as isoglosses for linguistic classification in dialectology is fully justified, these data introduce a bias into *phylogenetic classifications of languages*. In practice, the geographic bias in phylogenies may arise from implicitly giving preference to those features that fit the geographic distribution (used as a sort of “prior” in this case), while downplaying the features that are independent from geographic adjacency and language contact.

More generally, a less-than-perfect match between linguistic and geographic distances may have a more meaningful underlying cause than a simple bias of classification. Dialectometry deals with rather continuous linguistic landscapes where the change may spread in waves and for which it is possible to expect a cumulative increase of linguistic differences as a function of distance. The hypothetical situation of a maximum correlation of phylogenetic distances with geographic distances, on the other hand, can probably be described by a metaphor of an *explosion of a bombshell*. In this metaphor, pieces of the shell that were originally closest to each other would tend to split last and should lay closest to each other in space after a linguistic expansion has stabilized. We can safely assume that it is almost never the case. A scenario under which a language family starts from a point in space and then expands equally in all directions is unlikely. Phylogenetic divergence can be a corollary of ethnic splits and expansions, where groups of people move along trajectories that may depend on each others’ movements and on the landscape in a complex way. The divergence process is further complicated by cultural spreads of languages that are not associated with physical movement of the populations, but happen via assimilation. Instead of a static correlation, one is expected to come up with spatio-dynamic statistical models (possibly similar to those used in [49,50,89–92]) and test them against different phylogenetic hypotheses.

Dynamic models may account for complex trajectories as posited in ethnohistorical and ethnolinguistic approaches. Various scenarios that can lead to the observed geographic distributions of languages are suggested by Nichols [93], with many references to the languages of the Caucasus, including specifically Andic languages. She explains language history in terms of a spread directed uphill (or very rarely, a spread directed downhill, which she posits for Chechen), either resulting from a human migration or from a language shift. This spread uphill may lead to configurations such as the one that she calls ‘Burushaski distribution’, with an ‘older’ (in the sense of a longer stay *in situ*) language cut into discontinuous mountainous areas by a later spread of language into a valley between. She suggests as a possibility that several Andic uphills were cut off from their downhills and thus from each other by the spread of Avar. Alternatively, scenarios such as the one she calls ‘leapfrogging’ with an expanding language spreading uphill and leaving an older language on its way intact (this is what she suggests as a scenario for the Botlikh language, densely surrounded by Avar villages that she suspects have undergone language shift from one or more Andic languages to Avar). In other areas, such as Amazonia, scenarios that are different in details but similar in the nature of explanation are suggested, foregrounding language shift, as [3] for Arawakan or suggesting a combination of language shift with population migrations, as [4] for Tupian. Historical scenarios, including Nichols’ scenarios for Andic, remain, to our eyes, speculative, but may provide a tangible basis for further modeling of geographic distributions of languages.

To sum up, what comparison of different phylogenies in terms of geospatial correlation may tell us is which of the phylogenies are possibly more affected by language convergence or, eventually, by the author’s geographic bias in feature selection (causing a higher correlation with geography). In the case of Andic, the phylogenies that are presumably more immune to geography (quantitative, based on lexical data controlled for borrowings), are less correlated with geography than the phylogenies based on structural similarities or other, sometimes implicit, considerations. The comparison suggests that the latter are potentially more influenced by either language contact, or the author’s geographic bias towards the present-day spatial distribution of languages. We believe that in order to properly account for the geographic factor, one needs to use more complex approaches to building a lexicon-based quantitative phylogeny, such as dynamic models that take into account and compare different assumptions of human migrations and language shift. We hypothesize that such phylogenies would perform better in terms of correlation with the present-day geographic data. Testing this, however, is beyond the scope of this study.

## 6. Conclusion

In this study, our aim was to investigate the correlation between linguistic divergence and geographic distances. We investigated an area of high linguistic density, with 8 languages spoken in 77 villages packed in a small geographic space of about 6,600 km^2^ in the north of the Caucasus (Daghestan, Russia). This sample represents a family branch of an order comparable to that of Germanic or Romance, but, in geographic terms, is very close to, and even smaller than, the level at which dialectometry often operates. We have shown that the correlation between phylogeny and geography plays out at such a small scale. On the other hand, we could see that, opposite to what could be thought at a glance, the more assumedly precise the classification is (e.g. phylogenetic classification from a character-based evolutionary model vs. impressionistic grouping), the lower the correlation of geographic and linguistic distances is. This is due, we believe, to the fact that geographic distance cannot be used as the only or even the main predictor in modeling historical processes that shape linguistic divergence in the areas of high language density. Dynamic models of language spread rather than static models of language distribution can provide a more accurate account of language divergence. While the discussions of the possible scenarios in the literature remain, to our eyes, speculative, using them as priors in statistical analyses of the actual distributions of languages in terms of geographic distances certainly constitutes a plausible line of statistical approaches to modeling language density.

## Supporting information

S1 Data **S1 Fig: Correlation between GCD and travel cost (maximum, minimum, and symmetric)**.**S2 Fig. Koryakov’s topology: imputed tree with 77 villages (left) and simplified tree with 8 languages (right)****S3 Fig. Alekseev’s topology: imputed tree with 77 villages (left) and simplified tree with 8 languages (right)****S4 Fig. Schulze’s topology: imputed tree with 77 villages (left) and simplified tree with 8 languages (right)****S5 Fig. Filatov & Daniel’s topology: imputed tree with 77 villages (left) and simplified tree with 8 languages (right)****S6 Fig. Gudava’s topology: imputed tree with 77 villages (left) and simplified tree with 8 languages (right)****S7 Fig. Mudrak’s topology: imputed tree with 77 villages (left) and simplified tree with 8 languages (right)****S8 Fig. Flat topology: imputed tree with 77 villages (left) and simplified tree with 8 languages (right)****S9 Fig. Results for 77 villages (imputed trees)****S10 Fig. Kendall’s W for the correlation of each phylogeny with geography, using travel cost (compare with** Fig 6)**S11 Fig. Distributions of Kendall’s W for permuted topologies (1,000 permutations), using travel cost. Dashed lines represent the mean of the distribution, and red full lines represent the observed value for the topology, as red bars in S9 Fig. Compare with** Fig 7.**S12 Fig: Distributions of Kendall’s W for re-sampled village sets (1,000 permutations), using travel costs. Dashed lines represent the mean of the distribution, and red full lines represent the observed value for the topology, as red bars in S9 Fig. Compare with** Fig 8.**S1 Table. List of all villages and languages****S1 File. Comparison of geographic distances (travel cost vs. great circle distance)****S2 File. Discussion on imputed trees****S3 File. Code 0-data-cleaning.R****S4 File. Code 1-leastcostpath.R****S5 File. Code 2-phylogenies.R****S6 File. Code 3-correlations.R**
(ZIP)
