# Supplementary material for: Geography and language divergence: The case of Andic languages
Source: PLoS One. 2022 May 26;17(5):e0265460. doi: 10.1371/journal.pone.0265460 (PMC9135239; doi:10.1371/journal.pone.0265460)
Supplement: S1 Data — S1 Fig: Correlation between GCD and travel cost (maximum, minimum, and symmetric).S2 Fig. Koryakov’s topology: imputed tree with 77 villages (left) and simplified tree with 8 languages (right)S3 Fig. Alekseev’s topology: imputed tree with 77 villages (left) and simplified tree with 8 languages (right)S4 Fig. Schulze’s topology: imputed tree with 77 villages (left) and simplified tree with 8 languages (right)S5 Fig. Filatov & Daniel’s topology: imputed tree with 77 villages (left) and simplified tree with 8 languages (right)S6 Fig. Gudava’s topology: imputed tree with 77 villages (left) and simplified tree with 8 languages (right)S7 Fig. Mudrak’s topology: imputed tree with 77 villages (left) and simplified tree with 8 languages (right)S8 Fig. Flat topology: imputed tree with 77 villages (left) and simplified tree with 8 languages (right)S9 Fig. Results for 77 villages (imputed trees)S10 Fig. Kendall’s W for the correlation of each phylogeny with geography, using travel cost (compare with Fig 6)S11 Fig. Distributions of Kendall’s W for permuted topologies (1,000 permutations), using travel cost. Dashed lines represent the mean of the distribution, and red full lines represent the observed value for the topology, as red bars in S9 Fig. Compare with Fig 7.S12 Fig: Distributions of Kendall’s W for re-sampled village sets (1,000 permutations), using travel costs. Dashed lines represent the mean of the distribution, and red full lines represent the observed value for the topology, as red bars in S9 Fig. Compare with Fig 8.S1 Table. List of all villages and languagesS1 File. Comparison of geographic distances (travel cost vs. great circle distance)S2 File. Discussion on imputed treesS3 File. Code 0-data-cleaning.RS4 File. Code 1-leastcostpath.RS5 File. Code 2-phylogenies.RS6 File. Code 3-correlations.R (ZIP) [file pone.0265460.s001.zip › Supplementary_Information-new/S1_S2_File.docx]

**S1 File. Comparison of geographic distances (travel cost vs. great circle distance)**

We compare two different ways to measure geographic distances between each pair of points: The trivial GCD or straight line distance, and the path cost distance, considering the slope gradients.

Congruence Among Distance Matrices (CADM) analysis was conducted for comparing these two geographic distances (S1 Fig). Since the path cost distance is not symmetrical (it might be easier to go from A to B than from B to A, e.g. if the former is downhill) we separately calculated these correlations by using the maximum, minimum, and average travel distances between each pair of villages. It is clear from S1 Fig that i) choosing maximum, minimum, or average distances is practically irrelevant for these correlations, and ii) travel costs are highly correlated with GCD for our database. From i), we decide to use average travel costs for our following calculations. From ii), we see that using travel cost, although more realistic, will not sensibly change the results. We believe that this is due to the small area we are spanning, and these differences might become more clear when studying larger areas.

**S2 File. Discussion on imputed trees**

In this approach, we used imputed trees with all 77 villages in our database (see S2-S7 Figs for the imputed trees), from the qualitative classifications in Fig 4. We show the results in S9 Fig. We can see how, once more, Alekseev’s tree in [33] correlates better with geography, although most of the trees have a fairly good correlation of Kendall’s W around 0.75. The distinction between topologies becomes less perceptible, since they are identical to a great degree, using the same criterion of classifying villages that speak one given language together.
