# Supplementary figures and images for: Geography and language divergence: The case of Andic languages
Source: PLoS One. 2022 May 26;17(5):e0265460. doi: 10.1371/journal.pone.0265460 (PMC9135239; doi:10.1371/journal.pone.0265460)

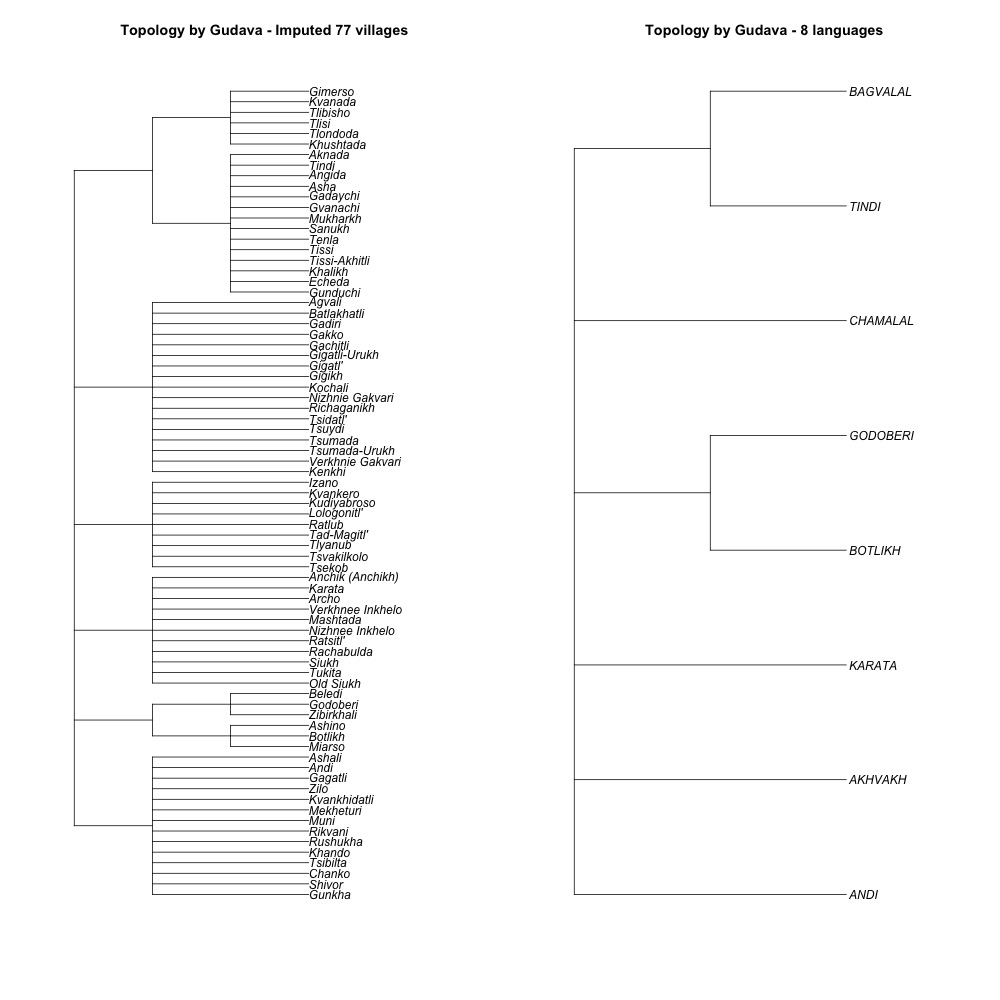

Supplement: S1 Data — S1 Fig: Correlation between GCD and travel cost (maximum, minimum, and symmetric).S2 Fig. Koryakov’s topology: imputed tree with 77 villages (left) and simplified tree with 8 languages (right)S3 Fig. Alekseev’s topology: imputed tree with 77 villages (left) and simplified tree with 8 languages (right)S4 Fig. Schulze’s topology: imputed tree with 77 villages (left) and simplified tree with 8 languages (right)S5 Fig. Filatov & Daniel’s topology: imputed tree with 77 villages (left) and simplified tree with 8 languages (right)S6 Fig. Gudava’s topology: imputed tree with 77 villages (left) and simplified tree with 8 languages (right)S7 Fig. Mudrak’s topology: imputed tree with 77 villages (left) and simplified tree with 8 languages (right)S8 Fig. Flat topology: imputed tree with 77 villages (left) and simplified tree with 8 languages (right)S9 Fig. Results for 77 villages (imputed trees)S10 Fig. Kendall’s W for the correlation of each phylogeny with geography, using travel cost (compare with Fig 6)S11 Fig. Distributions of Kendall’s W for permuted topologies (1,000 permutations), using travel cost. Dashed lines represent the mean of the distribution, and red full lines represent the observed value for the topology, as red bars in S9 Fig. Compare with Fig 7.S12 Fig: Distributions of Kendall’s W for re-sampled village sets (1,000 permutations), using travel costs. Dashed lines represent the mean of the distribution, and red full lines represent the observed value for the topology, as red bars in S9 Fig. Compare with Fig 8.S1 Table. List of all villages and languagesS1 File. Comparison of geographic distances (travel cost vs. great circle distance)S2 File. Discussion on imputed treesS3 File. Code 0-data-cleaning.RS4 File. Code 1-leastcostpath.RS5 File. Code 2-phylogenies.RS6 File. Code 3-correlations.R (ZIP) [file pone.0265460.s001.zip › Supplementary_Information-new/Sup_figs/S6Fig.jpg]

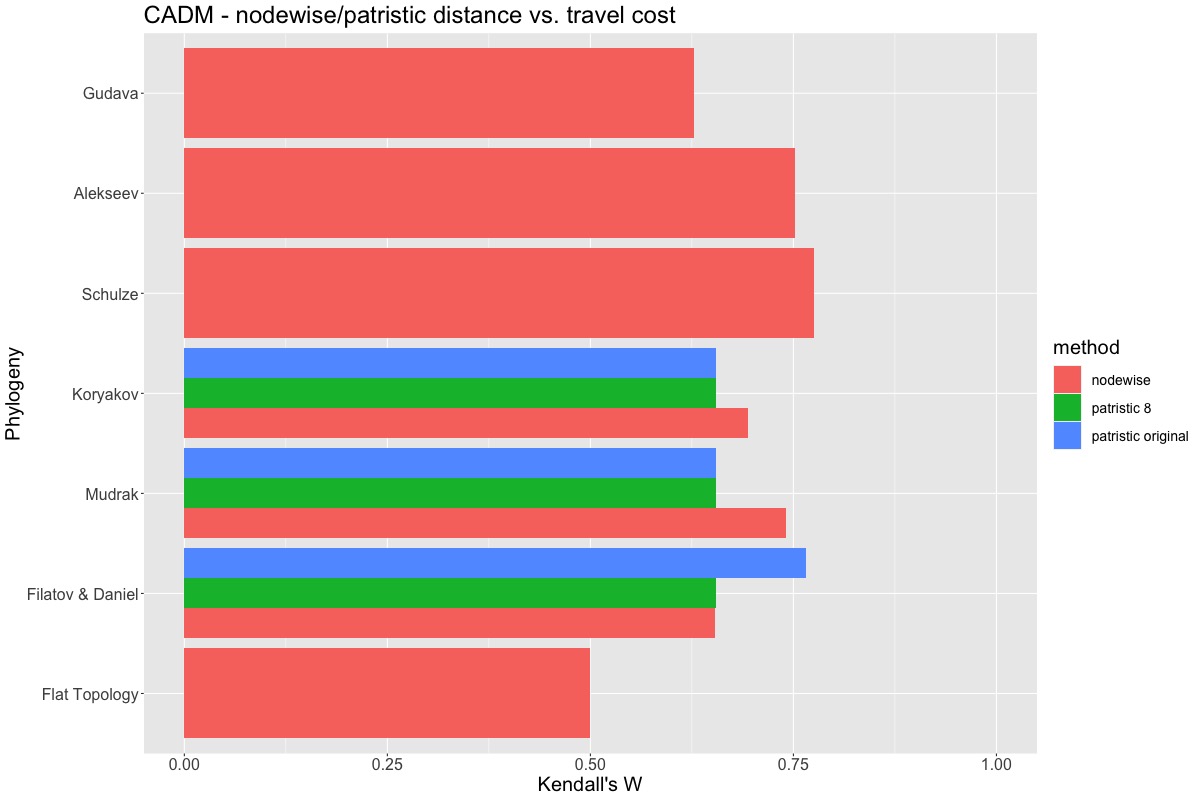

Supplement: S1 Data — S1 Fig: Correlation between GCD and travel cost (maximum, minimum, and symmetric).S2 Fig. Koryakov’s topology: imputed tree with 77 villages (left) and simplified tree with 8 languages (right)S3 Fig. Alekseev’s topology: imputed tree with 77 villages (left) and simplified tree with 8 languages (right)S4 Fig. Schulze’s topology: imputed tree with 77 villages (left) and simplified tree with 8 languages (right)S5 Fig. Filatov & Daniel’s topology: imputed tree with 77 villages (left) and simplified tree with 8 languages (right)S6 Fig. Gudava’s topology: imputed tree with 77 villages (left) and simplified tree with 8 languages (right)S7 Fig. Mudrak’s topology: imputed tree with 77 villages (left) and simplified tree with 8 languages (right)S8 Fig. Flat topology: imputed tree with 77 villages (left) and simplified tree with 8 languages (right)S9 Fig. Results for 77 villages (imputed trees)S10 Fig. Kendall’s W for the correlation of each phylogeny with geography, using travel cost (compare with Fig 6)S11 Fig. Distributions of Kendall’s W for permuted topologies (1,000 permutations), using travel cost. Dashed lines represent the mean of the distribution, and red full lines represent the observed value for the topology, as red bars in S9 Fig. Compare with Fig 7.S12 Fig: Distributions of Kendall’s W for re-sampled village sets (1,000 permutations), using travel costs. Dashed lines represent the mean of the distribution, and red full lines represent the observed value for the topology, as red bars in S9 Fig. Compare with Fig 8.S1 Table. List of all villages and languagesS1 File. Comparison of geographic distances (travel cost vs. great circle distance)S2 File. Discussion on imputed treesS3 File. Code 0-data-cleaning.RS4 File. Code 1-leastcostpath.RS5 File. Code 2-phylogenies.RS6 File. Code 3-correlations.R (ZIP) [file pone.0265460.s001.zip › Supplementary_Information-new/Sup_figs/S10Fig.jpg]

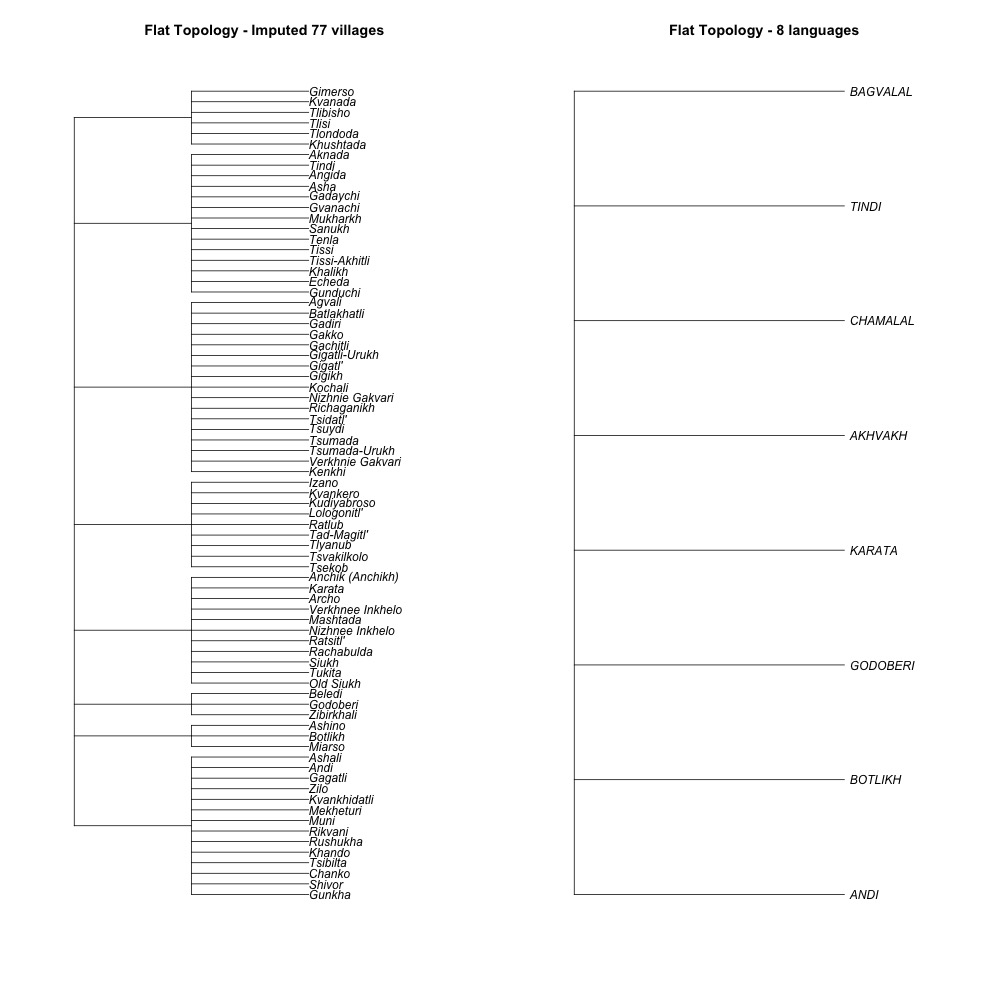

Supplement: S1 Data — S1 Fig: Correlation between GCD and travel cost (maximum, minimum, and symmetric).S2 Fig. Koryakov’s topology: imputed tree with 77 villages (left) and simplified tree with 8 languages (right)S3 Fig. Alekseev’s topology: imputed tree with 77 villages (left) and simplified tree with 8 languages (right)S4 Fig. Schulze’s topology: imputed tree with 77 villages (left) and simplified tree with 8 languages (right)S5 Fig. Filatov & Daniel’s topology: imputed tree with 77 villages (left) and simplified tree with 8 languages (right)S6 Fig. Gudava’s topology: imputed tree with 77 villages (left) and simplified tree with 8 languages (right)S7 Fig. Mudrak’s topology: imputed tree with 77 villages (left) and simplified tree with 8 languages (right)S8 Fig. Flat topology: imputed tree with 77 villages (left) and simplified tree with 8 languages (right)S9 Fig. Results for 77 villages (imputed trees)S10 Fig. Kendall’s W for the correlation of each phylogeny with geography, using travel cost (compare with Fig 6)S11 Fig. Distributions of Kendall’s W for permuted topologies (1,000 permutations), using travel cost. Dashed lines represent the mean of the distribution, and red full lines represent the observed value for the topology, as red bars in S9 Fig. Compare with Fig 7.S12 Fig: Distributions of Kendall’s W for re-sampled village sets (1,000 permutations), using travel costs. Dashed lines represent the mean of the distribution, and red full lines represent the observed value for the topology, as red bars in S9 Fig. Compare with Fig 8.S1 Table. List of all villages and languagesS1 File. Comparison of geographic distances (travel cost vs. great circle distance)S2 File. Discussion on imputed treesS3 File. Code 0-data-cleaning.RS4 File. Code 1-leastcostpath.RS5 File. Code 2-phylogenies.RS6 File. Code 3-correlations.R (ZIP) [file pone.0265460.s001.zip › Supplementary_Information-new/Sup_figs/S8Fig.jpg]

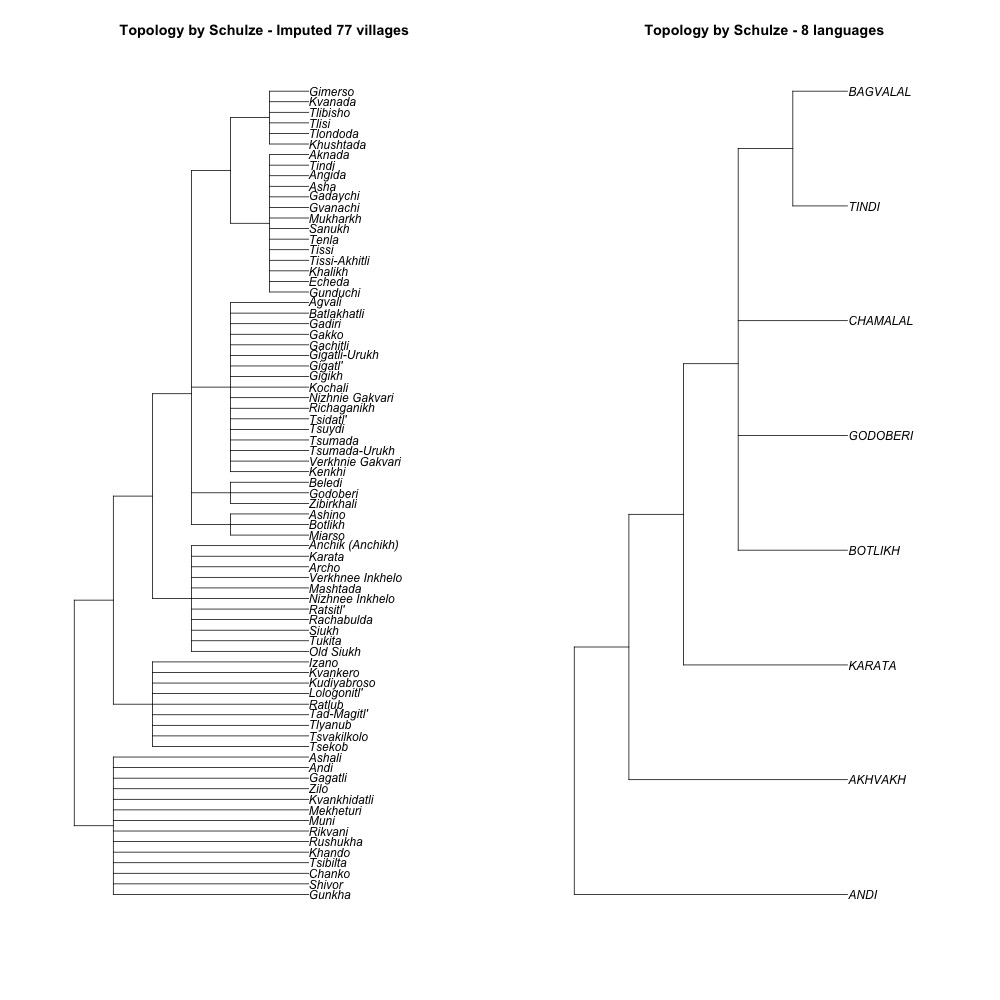

Supplement: S1 Data — S1 Fig: Correlation between GCD and travel cost (maximum, minimum, and symmetric).S2 Fig. Koryakov’s topology: imputed tree with 77 villages (left) and simplified tree with 8 languages (right)S3 Fig. Alekseev’s topology: imputed tree with 77 villages (left) and simplified tree with 8 languages (right)S4 Fig. Schulze’s topology: imputed tree with 77 villages (left) and simplified tree with 8 languages (right)S5 Fig. Filatov & Daniel’s topology: imputed tree with 77 villages (left) and simplified tree with 8 languages (right)S6 Fig. Gudava’s topology: imputed tree with 77 villages (left) and simplified tree with 8 languages (right)S7 Fig. Mudrak’s topology: imputed tree with 77 villages (left) and simplified tree with 8 languages (right)S8 Fig. Flat topology: imputed tree with 77 villages (left) and simplified tree with 8 languages (right)S9 Fig. Results for 77 villages (imputed trees)S10 Fig. Kendall’s W for the correlation of each phylogeny with geography, using travel cost (compare with Fig 6)S11 Fig. Distributions of Kendall’s W for permuted topologies (1,000 permutations), using travel cost. Dashed lines represent the mean of the distribution, and red full lines represent the observed value for the topology, as red bars in S9 Fig. Compare with Fig 7.S12 Fig: Distributions of Kendall’s W for re-sampled village sets (1,000 permutations), using travel costs. Dashed lines represent the mean of the distribution, and red full lines represent the observed value for the topology, as red bars in S9 Fig. Compare with Fig 8.S1 Table. List of all villages and languagesS1 File. Comparison of geographic distances (travel cost vs. great circle distance)S2 File. Discussion on imputed treesS3 File. Code 0-data-cleaning.RS4 File. Code 1-leastcostpath.RS5 File. Code 2-phylogenies.RS6 File. Code 3-correlations.R (ZIP) [file pone.0265460.s001.zip › Supplementary_Information-new/Sup_figs/S4Fig.jpg]

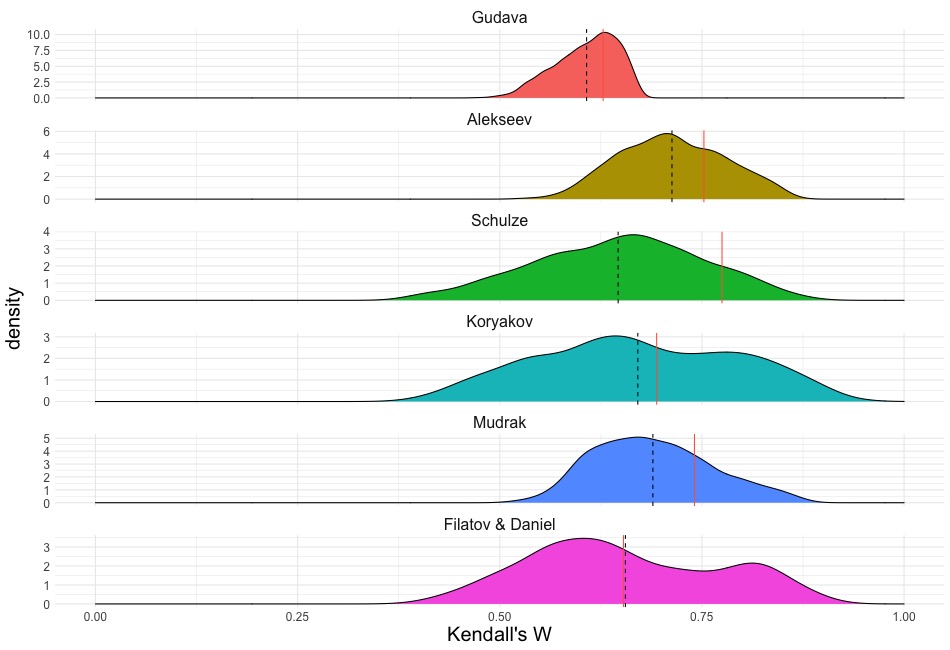

Supplement: S1 Data — S1 Fig: Correlation between GCD and travel cost (maximum, minimum, and symmetric).S2 Fig. Koryakov’s topology: imputed tree with 77 villages (left) and simplified tree with 8 languages (right)S3 Fig. Alekseev’s topology: imputed tree with 77 villages (left) and simplified tree with 8 languages (right)S4 Fig. Schulze’s topology: imputed tree with 77 villages (left) and simplified tree with 8 languages (right)S5 Fig. Filatov & Daniel’s topology: imputed tree with 77 villages (left) and simplified tree with 8 languages (right)S6 Fig. Gudava’s topology: imputed tree with 77 villages (left) and simplified tree with 8 languages (right)S7 Fig. Mudrak’s topology: imputed tree with 77 villages (left) and simplified tree with 8 languages (right)S8 Fig. Flat topology: imputed tree with 77 villages (left) and simplified tree with 8 languages (right)S9 Fig. Results for 77 villages (imputed trees)S10 Fig. Kendall’s W for the correlation of each phylogeny with geography, using travel cost (compare with Fig 6)S11 Fig. Distributions of Kendall’s W for permuted topologies (1,000 permutations), using travel cost. Dashed lines represent the mean of the distribution, and red full lines represent the observed value for the topology, as red bars in S9 Fig. Compare with Fig 7.S12 Fig: Distributions of Kendall’s W for re-sampled village sets (1,000 permutations), using travel costs. Dashed lines represent the mean of the distribution, and red full lines represent the observed value for the topology, as red bars in S9 Fig. Compare with Fig 8.S1 Table. List of all villages and languagesS1 File. Comparison of geographic distances (travel cost vs. great circle distance)S2 File. Discussion on imputed treesS3 File. Code 0-data-cleaning.RS4 File. Code 1-leastcostpath.RS5 File. Code 2-phylogenies.RS6 File. Code 3-correlations.R (ZIP) [file pone.0265460.s001.zip › Supplementary_Information-new/Sup_figs/S12Fig.jpg]

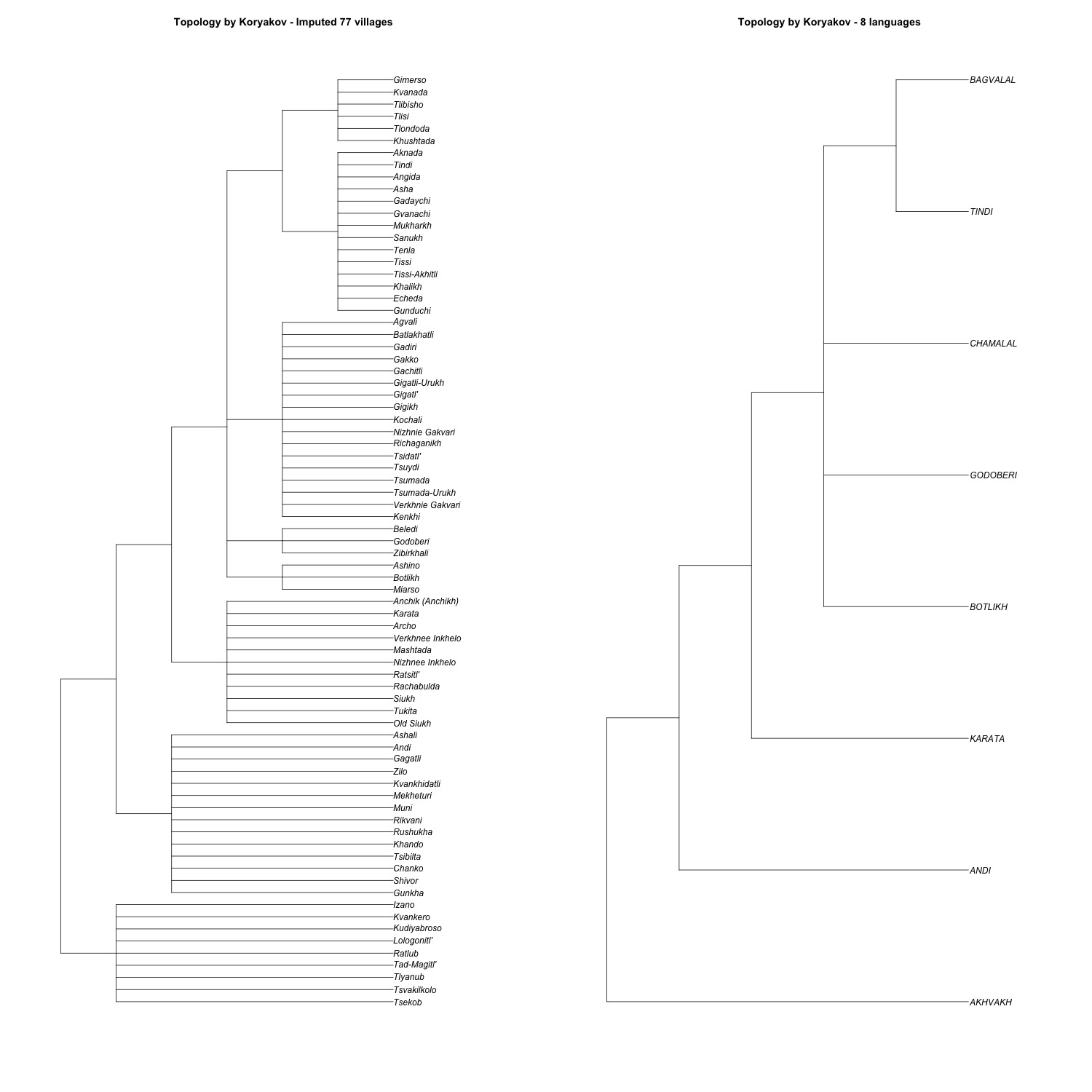

Supplement: S1 Data — S1 Fig: Correlation between GCD and travel cost (maximum, minimum, and symmetric).S2 Fig. Koryakov’s topology: imputed tree with 77 villages (left) and simplified tree with 8 languages (right)S3 Fig. Alekseev’s topology: imputed tree with 77 villages (left) and simplified tree with 8 languages (right)S4 Fig. Schulze’s topology: imputed tree with 77 villages (left) and simplified tree with 8 languages (right)S5 Fig. Filatov & Daniel’s topology: imputed tree with 77 villages (left) and simplified tree with 8 languages (right)S6 Fig. Gudava’s topology: imputed tree with 77 villages (left) and simplified tree with 8 languages (right)S7 Fig. Mudrak’s topology: imputed tree with 77 villages (left) and simplified tree with 8 languages (right)S8 Fig. Flat topology: imputed tree with 77 villages (left) and simplified tree with 8 languages (right)S9 Fig. Results for 77 villages (imputed trees)S10 Fig. Kendall’s W for the correlation of each phylogeny with geography, using travel cost (compare with Fig 6)S11 Fig. Distributions of Kendall’s W for permuted topologies (1,000 permutations), using travel cost. Dashed lines represent the mean of the distribution, and red full lines represent the observed value for the topology, as red bars in S9 Fig. Compare with Fig 7.S12 Fig: Distributions of Kendall’s W for re-sampled village sets (1,000 permutations), using travel costs. Dashed lines represent the mean of the distribution, and red full lines represent the observed value for the topology, as red bars in S9 Fig. Compare with Fig 8.S1 Table. List of all villages and languagesS1 File. Comparison of geographic distances (travel cost vs. great circle distance)S2 File. Discussion on imputed treesS3 File. Code 0-data-cleaning.RS4 File. Code 1-leastcostpath.RS5 File. Code 2-phylogenies.RS6 File. Code 3-correlations.R (ZIP) [file pone.0265460.s001.zip › Supplementary_Information-new/Sup_figs/S2Fig.jpg]

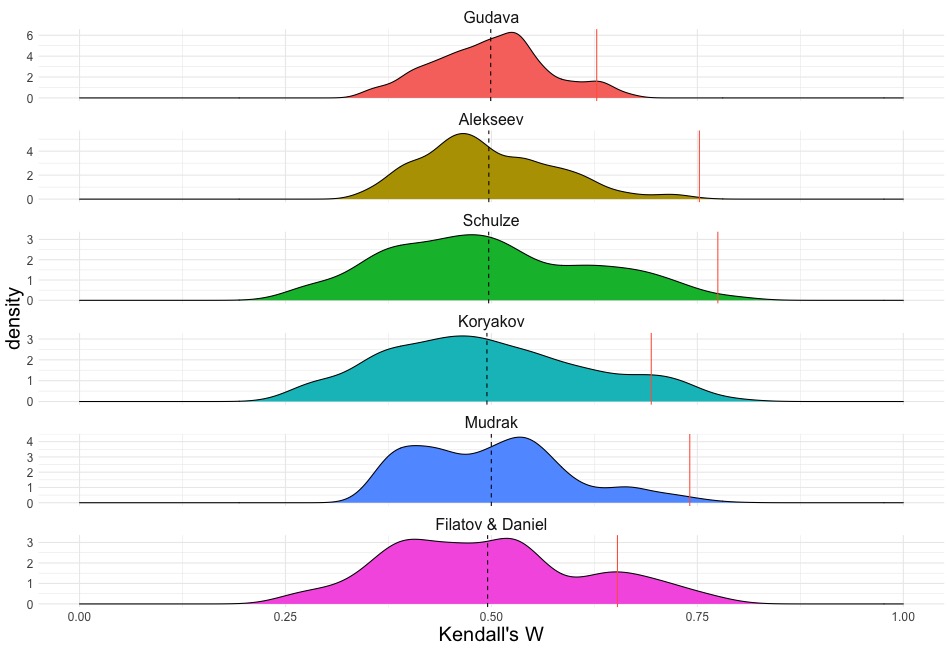

Supplement: S1 Data — S1 Fig: Correlation between GCD and travel cost (maximum, minimum, and symmetric).S2 Fig. Koryakov’s topology: imputed tree with 77 villages (left) and simplified tree with 8 languages (right)S3 Fig. Alekseev’s topology: imputed tree with 77 villages (left) and simplified tree with 8 languages (right)S4 Fig. Schulze’s topology: imputed tree with 77 villages (left) and simplified tree with 8 languages (right)S5 Fig. Filatov & Daniel’s topology: imputed tree with 77 villages (left) and simplified tree with 8 languages (right)S6 Fig. Gudava’s topology: imputed tree with 77 villages (left) and simplified tree with 8 languages (right)S7 Fig. Mudrak’s topology: imputed tree with 77 villages (left) and simplified tree with 8 languages (right)S8 Fig. Flat topology: imputed tree with 77 villages (left) and simplified tree with 8 languages (right)S9 Fig. Results for 77 villages (imputed trees)S10 Fig. Kendall’s W for the correlation of each phylogeny with geography, using travel cost (compare with Fig 6)S11 Fig. Distributions of Kendall’s W for permuted topologies (1,000 permutations), using travel cost. Dashed lines represent the mean of the distribution, and red full lines represent the observed value for the topology, as red bars in S9 Fig. Compare with Fig 7.S12 Fig: Distributions of Kendall’s W for re-sampled village sets (1,000 permutations), using travel costs. Dashed lines represent the mean of the distribution, and red full lines represent the observed value for the topology, as red bars in S9 Fig. Compare with Fig 8.S1 Table. List of all villages and languagesS1 File. Comparison of geographic distances (travel cost vs. great circle distance)S2 File. Discussion on imputed treesS3 File. Code 0-data-cleaning.RS4 File. Code 1-leastcostpath.RS5 File. Code 2-phylogenies.RS6 File. Code 3-correlations.R (ZIP) [file pone.0265460.s001.zip › Supplementary_Information-new/Sup_figs/S11Fig.jpg]

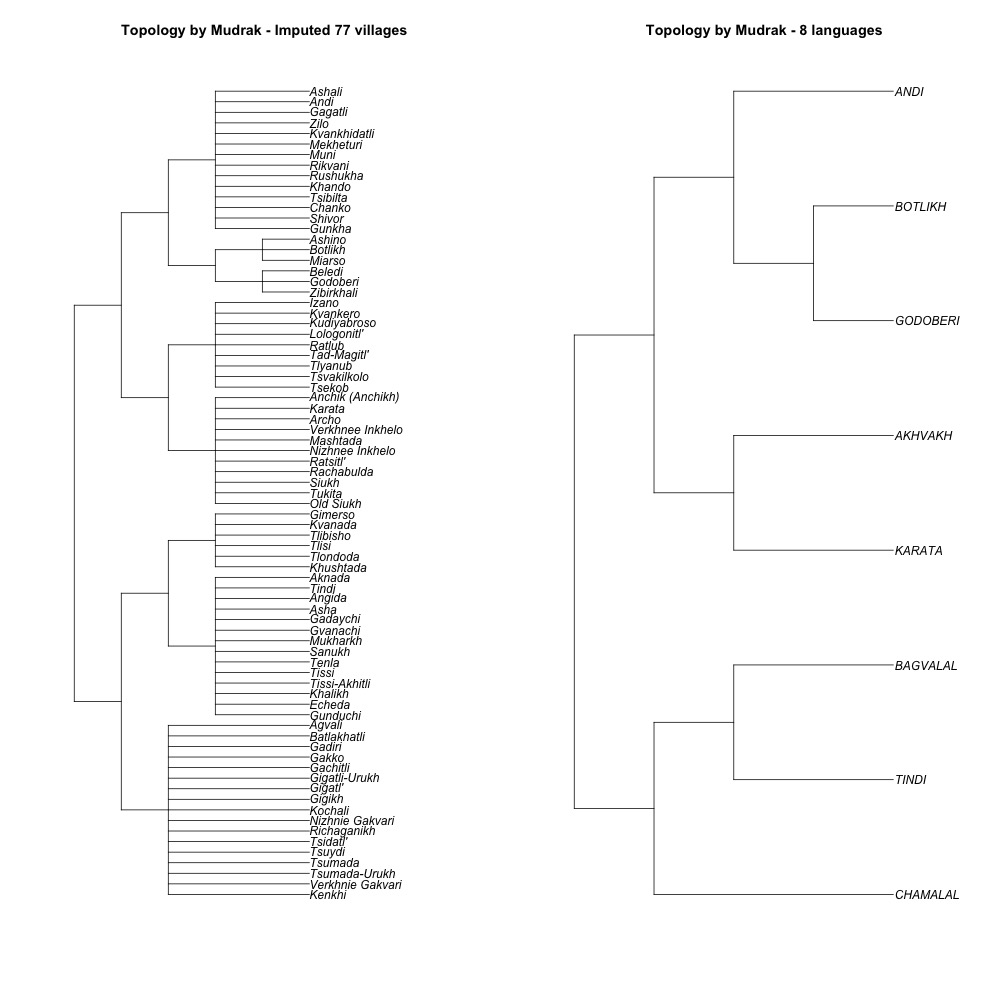

Supplement: S1 Data — S1 Fig: Correlation between GCD and travel cost (maximum, minimum, and symmetric).S2 Fig. Koryakov’s topology: imputed tree with 77 villages (left) and simplified tree with 8 languages (right)S3 Fig. Alekseev’s topology: imputed tree with 77 villages (left) and simplified tree with 8 languages (right)S4 Fig. Schulze’s topology: imputed tree with 77 villages (left) and simplified tree with 8 languages (right)S5 Fig. Filatov & Daniel’s topology: imputed tree with 77 villages (left) and simplified tree with 8 languages (right)S6 Fig. Gudava’s topology: imputed tree with 77 villages (left) and simplified tree with 8 languages (right)S7 Fig. Mudrak’s topology: imputed tree with 77 villages (left) and simplified tree with 8 languages (right)S8 Fig. Flat topology: imputed tree with 77 villages (left) and simplified tree with 8 languages (right)S9 Fig. Results for 77 villages (imputed trees)S10 Fig. Kendall’s W for the correlation of each phylogeny with geography, using travel cost (compare with Fig 6)S11 Fig. Distributions of Kendall’s W for permuted topologies (1,000 permutations), using travel cost. Dashed lines represent the mean of the distribution, and red full lines represent the observed value for the topology, as red bars in S9 Fig. Compare with Fig 7.S12 Fig: Distributions of Kendall’s W for re-sampled village sets (1,000 permutations), using travel costs. Dashed lines represent the mean of the distribution, and red full lines represent the observed value for the topology, as red bars in S9 Fig. Compare with Fig 8.S1 Table. List of all villages and languagesS1 File. Comparison of geographic distances (travel cost vs. great circle distance)S2 File. Discussion on imputed treesS3 File. Code 0-data-cleaning.RS4 File. Code 1-leastcostpath.RS5 File. Code 2-phylogenies.RS6 File. Code 3-correlations.R (ZIP) [file pone.0265460.s001.zip › Supplementary_Information-new/Sup_figs/S7Fig.jpg]

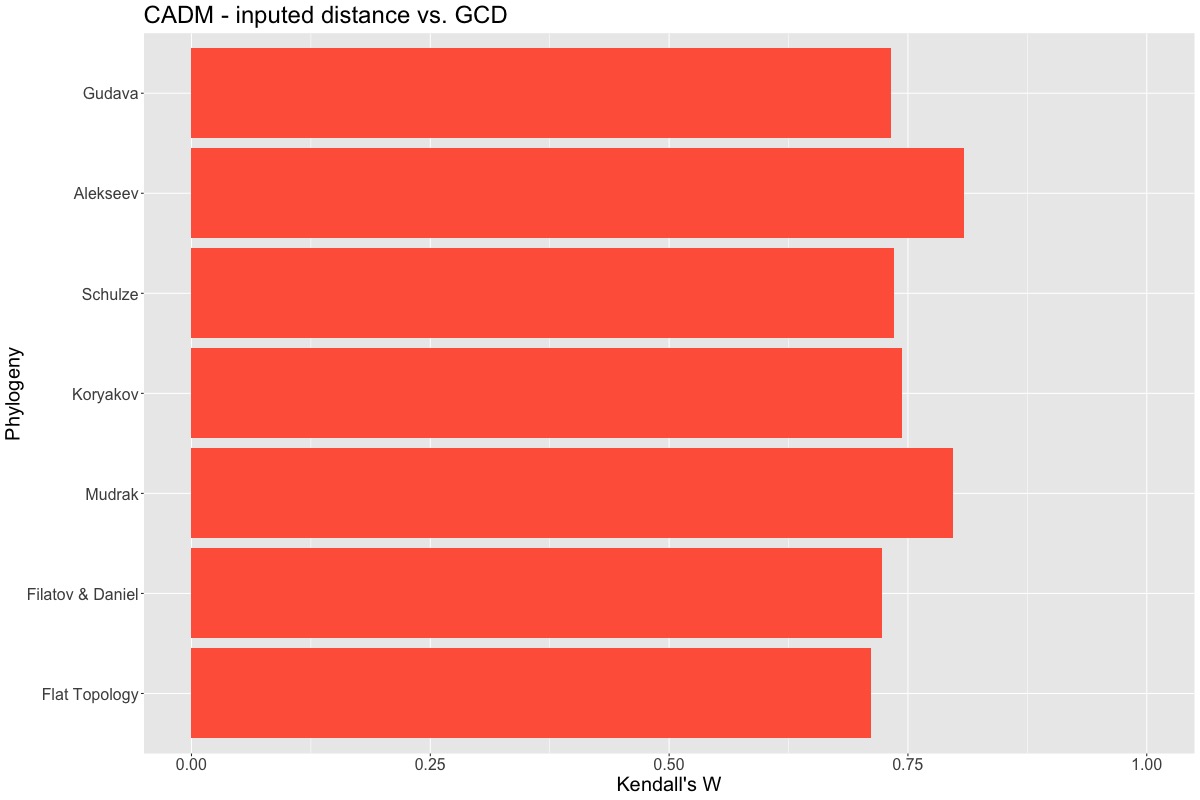

Supplement: S1 Data — S1 Fig: Correlation between GCD and travel cost (maximum, minimum, and symmetric).S2 Fig. Koryakov’s topology: imputed tree with 77 villages (left) and simplified tree with 8 languages (right)S3 Fig. Alekseev’s topology: imputed tree with 77 villages (left) and simplified tree with 8 languages (right)S4 Fig. Schulze’s topology: imputed tree with 77 villages (left) and simplified tree with 8 languages (right)S5 Fig. Filatov & Daniel’s topology: imputed tree with 77 villages (left) and simplified tree with 8 languages (right)S6 Fig. Gudava’s topology: imputed tree with 77 villages (left) and simplified tree with 8 languages (right)S7 Fig. Mudrak’s topology: imputed tree with 77 villages (left) and simplified tree with 8 languages (right)S8 Fig. Flat topology: imputed tree with 77 villages (left) and simplified tree with 8 languages (right)S9 Fig. Results for 77 villages (imputed trees)S10 Fig. Kendall’s W for the correlation of each phylogeny with geography, using travel cost (compare with Fig 6)S11 Fig. Distributions of Kendall’s W for permuted topologies (1,000 permutations), using travel cost. Dashed lines represent the mean of the distribution, and red full lines represent the observed value for the topology, as red bars in S9 Fig. Compare with Fig 7.S12 Fig: Distributions of Kendall’s W for re-sampled village sets (1,000 permutations), using travel costs. Dashed lines represent the mean of the distribution, and red full lines represent the observed value for the topology, as red bars in S9 Fig. Compare with Fig 8.S1 Table. List of all villages and languagesS1 File. Comparison of geographic distances (travel cost vs. great circle distance)S2 File. Discussion on imputed treesS3 File. Code 0-data-cleaning.RS4 File. Code 1-leastcostpath.RS5 File. Code 2-phylogenies.RS6 File. Code 3-correlations.R (ZIP) [file pone.0265460.s001.zip › Supplementary_Information-new/Sup_figs/S9Fig.jpg]

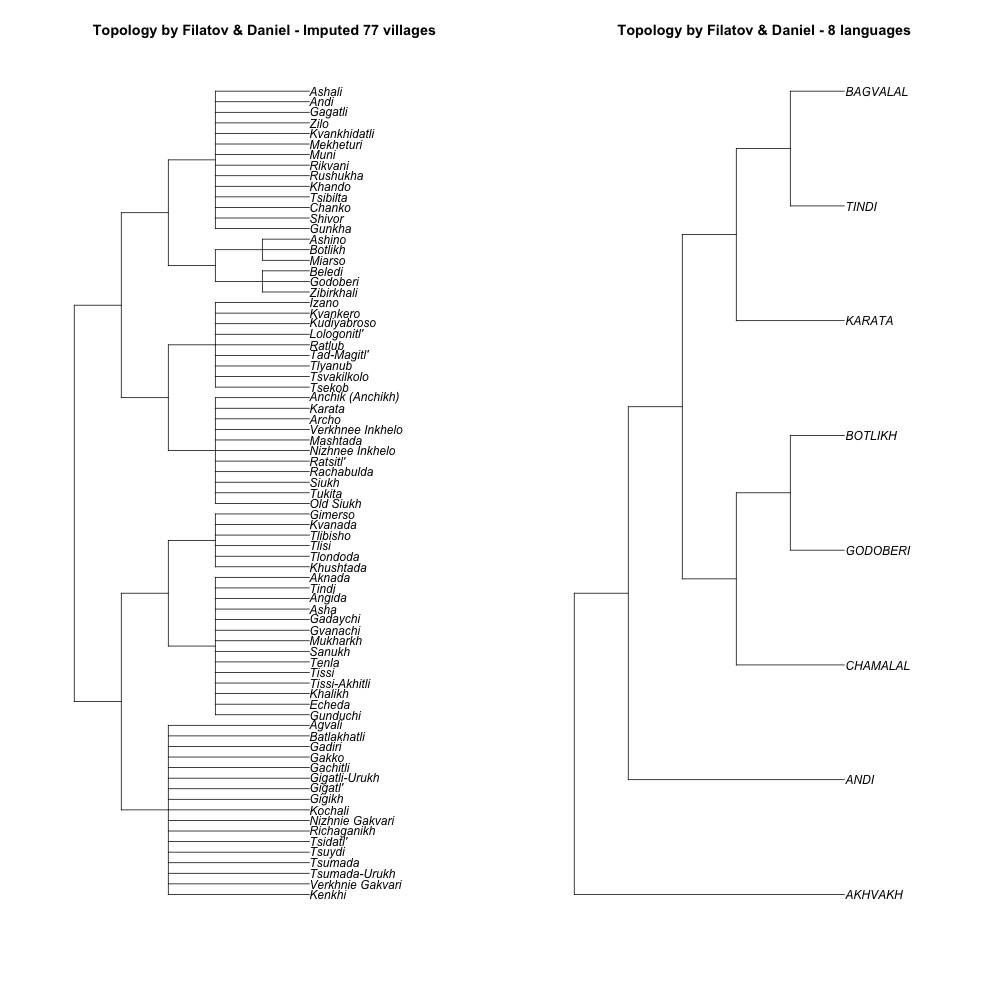

Supplement: S1 Data — S1 Fig: Correlation between GCD and travel cost (maximum, minimum, and symmetric).S2 Fig. Koryakov’s topology: imputed tree with 77 villages (left) and simplified tree with 8 languages (right)S3 Fig. Alekseev’s topology: imputed tree with 77 villages (left) and simplified tree with 8 languages (right)S4 Fig. Schulze’s topology: imputed tree with 77 villages (left) and simplified tree with 8 languages (right)S5 Fig. Filatov & Daniel’s topology: imputed tree with 77 villages (left) and simplified tree with 8 languages (right)S6 Fig. Gudava’s topology: imputed tree with 77 villages (left) and simplified tree with 8 languages (right)S7 Fig. Mudrak’s topology: imputed tree with 77 villages (left) and simplified tree with 8 languages (right)S8 Fig. Flat topology: imputed tree with 77 villages (left) and simplified tree with 8 languages (right)S9 Fig. Results for 77 villages (imputed trees)S10 Fig. Kendall’s W for the correlation of each phylogeny with geography, using travel cost (compare with Fig 6)S11 Fig. Distributions of Kendall’s W for permuted topologies (1,000 permutations), using travel cost. Dashed lines represent the mean of the distribution, and red full lines represent the observed value for the topology, as red bars in S9 Fig. Compare with Fig 7.S12 Fig: Distributions of Kendall’s W for re-sampled village sets (1,000 permutations), using travel costs. Dashed lines represent the mean of the distribution, and red full lines represent the observed value for the topology, as red bars in S9 Fig. Compare with Fig 8.S1 Table. List of all villages and languagesS1 File. Comparison of geographic distances (travel cost vs. great circle distance)S2 File. Discussion on imputed treesS3 File. Code 0-data-cleaning.RS4 File. Code 1-leastcostpath.RS5 File. Code 2-phylogenies.RS6 File. Code 3-correlations.R (ZIP) [file pone.0265460.s001.zip › Supplementary_Information-new/Sup_figs/S5Fig.jpg]

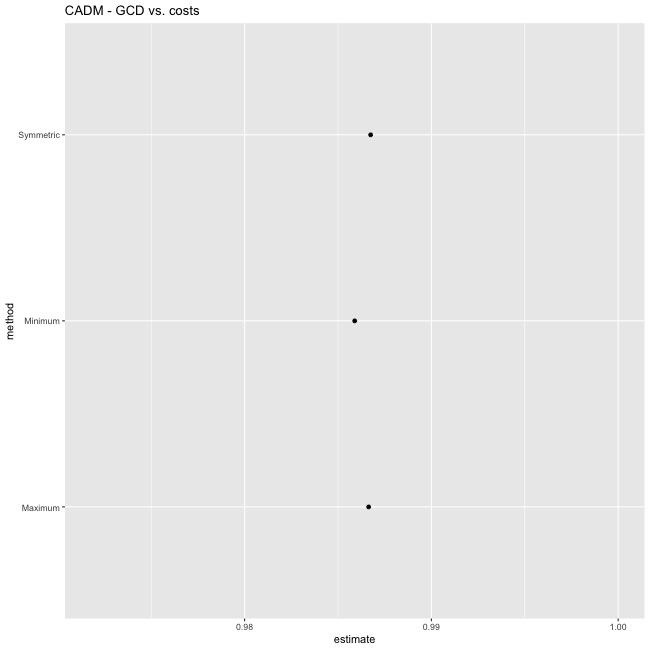

Supplement: S1 Data — S1 Fig: Correlation between GCD and travel cost (maximum, minimum, and symmetric).S2 Fig. Koryakov’s topology: imputed tree with 77 villages (left) and simplified tree with 8 languages (right)S3 Fig. Alekseev’s topology: imputed tree with 77 villages (left) and simplified tree with 8 languages (right)S4 Fig. Schulze’s topology: imputed tree with 77 villages (left) and simplified tree with 8 languages (right)S5 Fig. Filatov & Daniel’s topology: imputed tree with 77 villages (left) and simplified tree with 8 languages (right)S6 Fig. Gudava’s topology: imputed tree with 77 villages (left) and simplified tree with 8 languages (right)S7 Fig. Mudrak’s topology: imputed tree with 77 villages (left) and simplified tree with 8 languages (right)S8 Fig. Flat topology: imputed tree with 77 villages (left) and simplified tree with 8 languages (right)S9 Fig. Results for 77 villages (imputed trees)S10 Fig. Kendall’s W for the correlation of each phylogeny with geography, using travel cost (compare with Fig 6)S11 Fig. Distributions of Kendall’s W for permuted topologies (1,000 permutations), using travel cost. Dashed lines represent the mean of the distribution, and red full lines represent the observed value for the topology, as red bars in S9 Fig. Compare with Fig 7.S12 Fig: Distributions of Kendall’s W for re-sampled village sets (1,000 permutations), using travel costs. Dashed lines represent the mean of the distribution, and red full lines represent the observed value for the topology, as red bars in S9 Fig. Compare with Fig 8.S1 Table. List of all villages and languagesS1 File. Comparison of geographic distances (travel cost vs. great circle distance)S2 File. Discussion on imputed treesS3 File. Code 0-data-cleaning.RS4 File. Code 1-leastcostpath.RS5 File. Code 2-phylogenies.RS6 File. Code 3-correlations.R (ZIP) [file pone.0265460.s001.zip › Supplementary_Information-new/Sup_figs/S1Fig.jpg]

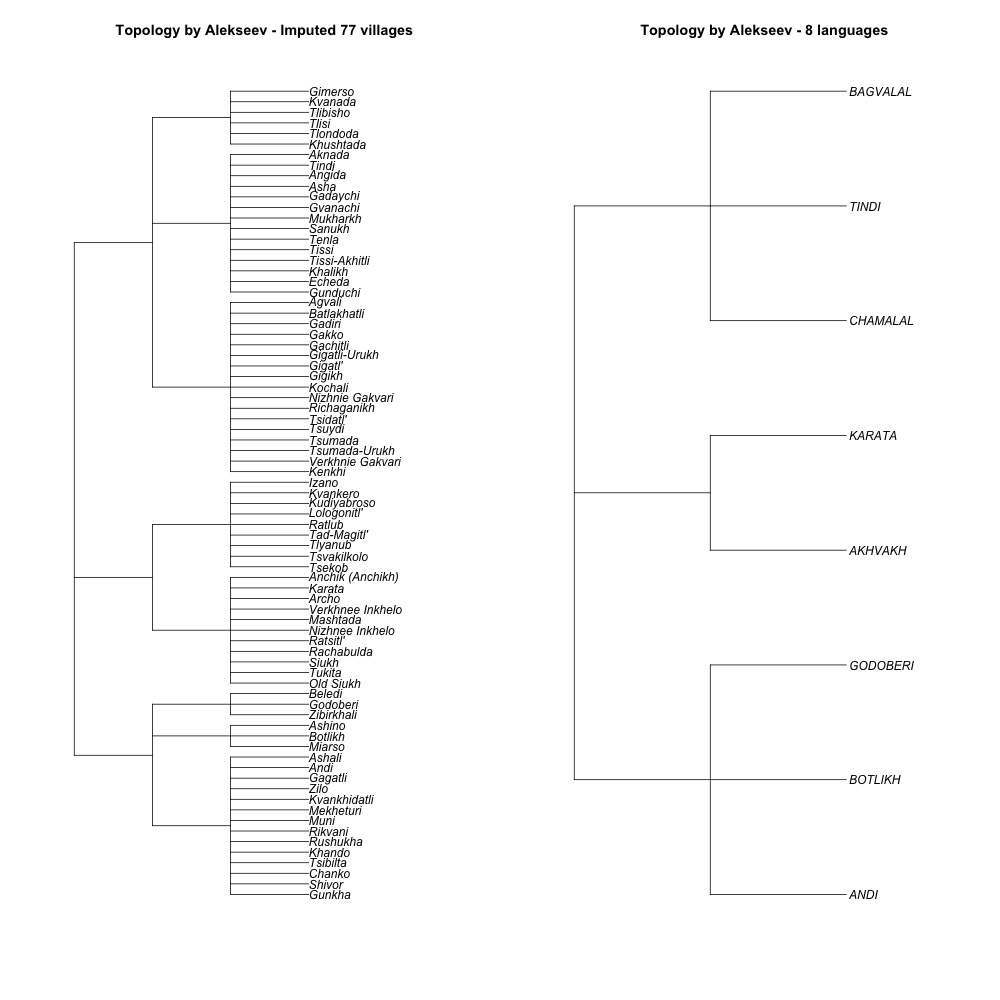

Supplement: S1 Data — S1 Fig: Correlation between GCD and travel cost (maximum, minimum, and symmetric).S2 Fig. Koryakov’s topology: imputed tree with 77 villages (left) and simplified tree with 8 languages (right)S3 Fig. Alekseev’s topology: imputed tree with 77 villages (left) and simplified tree with 8 languages (right)S4 Fig. Schulze’s topology: imputed tree with 77 villages (left) and simplified tree with 8 languages (right)S5 Fig. Filatov & Daniel’s topology: imputed tree with 77 villages (left) and simplified tree with 8 languages (right)S6 Fig. Gudava’s topology: imputed tree with 77 villages (left) and simplified tree with 8 languages (right)S7 Fig. Mudrak’s topology: imputed tree with 77 villages (left) and simplified tree with 8 languages (right)S8 Fig. Flat topology: imputed tree with 77 villages (left) and simplified tree with 8 languages (right)S9 Fig. Results for 77 villages (imputed trees)S10 Fig. Kendall’s W for the correlation of each phylogeny with geography, using travel cost (compare with Fig 6)S11 Fig. Distributions of Kendall’s W for permuted topologies (1,000 permutations), using travel cost. Dashed lines represent the mean of the distribution, and red full lines represent the observed value for the topology, as red bars in S9 Fig. Compare with Fig 7.S12 Fig: Distributions of Kendall’s W for re-sampled village sets (1,000 permutations), using travel costs. Dashed lines represent the mean of the distribution, and red full lines represent the observed value for the topology, as red bars in S9 Fig. Compare with Fig 8.S1 Table. List of all villages and languagesS1 File. Comparison of geographic distances (travel cost vs. great circle distance)S2 File. Discussion on imputed treesS3 File. Code 0-data-cleaning.RS4 File. Code 1-leastcostpath.RS5 File. Code 2-phylogenies.RS6 File. Code 3-correlations.R (ZIP) [file pone.0265460.s001.zip › Supplementary_Information-new/Sup_figs/S3Fig.jpg]
